# Exome Sequencing Reveals a Phenotype Modifying Variant in *ZNF528* in Primary Osteoporosis With a *COL1A2* Deletion

**DOI:** 10.1002/jbmr.4145

**Published:** 2020-08-26

**Authors:** Sini Skarp, Ji‐Han Xia, Qin Zhang, Marika Löija, Alice Costantini, Lloyd W Ruddock, Outi Mäkitie, Gong‐Hong Wei, Minna Männikkö

**Affiliations:** ^1^ Infrastructure for Population Studies Northern Finland Birth Cohorts, Faculty of Medicine, University of Oulu Oulu Finland; ^2^ Faculty of Biochemistry and Molecular Medicine University of Oulu Oulu Finland; ^3^ Center for Life Course Health Research Faculty of Medicine, University of Oulu Oulu Finland; ^4^ Biocenter Oulu University of Oulu Oulu Finland; ^5^ Department of Molecular Medicine and Surgery and Center for Molecular Medicine Karolinska Institutet/Stockholm Stockholm Sweden; ^6^ Department of Clinical Genetics Karolinska University Hospital Stockholm Sweden; ^7^ Children's Hospital and Pediatric Research Center University of Helsinki and Helsinki University Hospital Helsinki Finland; ^8^ Folkhälsan Research Center Genetics Research Program Helsinki Finland; ^9^ Research Program for Clinical and Molecular Metabolism Faculty of Medicine, University of Helsinki Helsinki Finland; ^10^ Department of Biochemistry and Molecular Biology School of Basic Medical Sciences and Zhongshan Hospital, Fudan University Shanghai China

**Keywords:** COL1A2, EXOME SEQUENCING, PRIMARY OSTEOPOROSIS, TRANSCRIPTION FACTOR, ZNF528

## Abstract

We studied a family with severe primary osteoporosis carrying a heterozygous p.Arg8Phefs*14 deletion in COL1A2, leading to haploinsufficiency. Three affected individuals carried the mutation and presented nearly identical spinal fractures but lacked other typical features of either osteogenesis imperfecta or Ehlers‐Danlos syndrome. Although mutations leading to haploinsufficiency in COL1A2 are rare, mutations in COL1A1 that lead to less protein typically result in a milder phenotype. We hypothesized that other genetic factors may contribute to the severe phenotype in this family. We performed whole‐exome sequencing in five family members and identified in all three affected individuals a rare nonsense variant (c.1282C > T/p.Arg428*, rs150257846) in ZNF528. We studied the effect of the variant using qPCR and Western blot and its subcellular localization with immunofluorescence. Our results indicate production of a truncated ZNF528 protein that locates in the cell nucleus as per the wild‐type protein. ChIP and RNA sequencing analyses on ZNF528 and ZNF528‐c.1282C > T indicated that ZNF528 binding sites are linked to pathways and genes regulating bone morphology. Compared with the wild type, ZNF528‐c.1282C > T showed a global shift in genomic binding profile and pathway enrichment, possibly contributing to the pathophysiology of primary osteoporosis. We identified five putative target genes for ZNF528 and showed that the expression of these genes is altered in patient cells. In conclusion, the variant leads to expression of truncated ZNF528 and a global change of its genomic occupancy, which in turn may lead to altered expression of target genes. ZNF528 is a novel candidate gene for bone disorders and may function as a transcriptional regulator in pathways affecting bone morphology and contribute to the phenotype of primary osteoporosis in this family together with the COL1A2 deletion. © 2020 The Authors. *Journal of Bone and Mineral Research* published by Wiley Periodicals LLC on behalf of American Society for Bone and Mineral Research (ASBMR).

## Introduction

Osteogenesis imperfecta (OI) is a rare genetic disorder characterized by low bone mass and bone fragility.^(^ ^1^ ^)^ Classical OI types are inherited in an autosomal dominant manner, but autosomal recessive forms have also been reported.^(^ ^1^, ^2^, ^3^, ^4^ ^)^ Clinical manifestations of OI range from mild symptoms to severe bone deformities and neonatal lethality.^(^ ^5^ ^)^ Most OI cases are caused by mutations in the genes encoding for collagen I alpha chains, *COL1A1* and *COL1A2*.^(^ ^6^ ^)^ Other OI‐causing mutations are often in genes involved in the synthesis, posttranslational modification, trafficking, or secretion of collagen I.^(^ ^7^, ^8^, ^9^, ^10^, ^11^, ^12^ ^)^ In addition, mutations in the zinc‐finger transcription factor Sp7 (a.k.a. Osterix) required for osteoblast differentiation and bone formation have been reported to cause an autosomal recessive form of OI.^(^ ^13^, ^14^, ^15^ ^)^

Collagen I is a triple helical protein formed of two alpha 1 and one alpha 2 chains. The triple helical structure is formed from a repeating region where every third amino acid is a glycine.^(^ ^16^ ^)^ Mutations affecting this region disturb the interaction with other collagen chains and lead to synthesis of abnormally structured collagen I.^(^ ^16^ ^)^ This is a common mutation type in OI types II to IV, where clinical phenotype ranges from moderate to lethal.^(^ ^6^ ^)^ Mutations affecting the N‐propeptide cleavage site cause another connective tissue disorder called Ehlers‐Danlos syndrome (EDS).^(^ ^17^ ^)^ EDS is a group of disorders characterized by fragility of connective tissue, atrophic scarring, skin hyperextensibility, hypermobility of joints, and abnormal bruising. The EDS type caused by mutations in the collagen I encoding genes is a rare autosomal dominant arthrochalasic form characterized by serious joint hypermobility, hip dysplasia, skin hyperextensibility, bruising, arthropic scars, kyphoscoliosis, and osteopenia.^(^ ^18^ ^)^

The mildest OI, type I, is often caused by heterozygous mutations in *COL1A1*, leading to nonsense mediated decay and haploinsufficiency.^(^ ^6^ ^)^ Homozygous or compound heterozygous mutations causing complete lack of collagen I alpha 2 chain typically result in EDS with a defect in cardiac valve,^(^ ^19^, ^20^, ^21^ ^)^ but in some cases they have been associated with moderate or severe OI.^(^ ^22^, ^23^, ^24^, ^25^ ^)^ In addition, a mixed EDS/OI phenotype has been described.^(^ ^26^ ^)^ There is no clear understanding of the mechanisms leading to differential outcomes of the lack of collagen I alpha2 chain. It has been suggested that in some OI cases the observed mutations may not lead to complete lack of *COL1A2* and some misfolded protein might accumulate in the cell.^(^ ^22^ ^)^ Mutations leading to haploinsufficiency are very rare in *COL1A2*.^(^ ^27^, ^28^ ^)^ In *COL1A1* and in *COL2A1*, coding for collagen II, mutations leading to reduced protein, rather than defected protein, typically result in milder phenotypes.^(^ ^6^, ^29^ ^)^

Although there is some correlation between collagen I mutation types and different phenotypes, this relationship is complex and identical mutations in the collagen I genes may result in phenotypic variability in OI even within families with identical mutations.^30^, ^31^ ^)^ Other genetic factors are likely to contribute to this variability. However, no such modifiers have previously been identified.

Heterozygous p.Arg8Phefs^*^14 deletion in *COL1A2* leading to nonsense mediated decay was recently identified in a Finnish family with primary osteoporosis.^(^ ^32^ ^)^ Three affected individuals carried the mutation and presented nearly identical spinal fractures in radiographs, but they lacked otherwise typical features for both OI and EDS, such as blue sclerae, dentinogenesis imperfecta, hypermobility, muscle weakness, deformities, and short stature. In this family, all affected individuals had severe early onset primary osteoporosis with low bone mineral density (BMD) and several spinal fractures since childhood. We hypothesized that other genetic factors may contribute to the severe phenotype and thus performed whole‐exome sequencing in five members of the family. We identified a pathogenic variant in ZNF528 transcription factor likely to affect the phenotype by altering the expression of its target genes in pathways affecting bone morphology.

## Materials and Methods

Methods are described in more detail in the Supplemental Materials and Methods.

### Patients

Affected individuals in the family, the father and two sons, had suffered from multiple compression fractures of the vertebrae. The pedigree of the family is presented in Supplemental Fig. S1. By the age of 30 years, the father had already been diagnosed with multiple compression fractures and he had lost 5 cm of adult height. The sons had been diagnosed with compression fractures in childhood, at the age of 8 and 12 years (Supplemental Fig. S2). They also had persistently low lumbar spine BMD but normal BMD at the proximal hip (Supplemental Fig. S2; Supplemental Table S1). The mother, third son, and three siblings of the father were unaffected. The family has previously been described in more detail.^(^ ^32^ ^)^

DNA samples were available from all eight family members (three affected, five unaffected). Genomic DNA was extracted from EDTA blood samples using standard procedures. DNA sample quality and quantity were characterized by NanoDrop measurement. Primary skin fibroblasts were available from two affected family members (I2 and II2, Supplemental Fig. S1) and three unrelated controls. This study was approved by the Ethics Committee of the Hospital District of Helsinki and Uusimaa, and family members gave their informed written consent.

### Whole‐exome data analysis

DNA samples from five family members (Supplemental Fig. S1) were studied using whole‐exome sequencing. DNA was fragmented and enriched at the BGI (http://www.genomics.cn/en/index) by using NimbleGen SeqCap EZ Human Exome v3.0 kit. The enriched DNA was subjected to Illumina (San Diego, CA, USA) HiSeq sequencing. Clean reads were then aligned with the reference genome (hg19) and quality controlled according to the BGI Bioinformatics (Cambridge, MA, USA) pipeline.

Private or rare (minor allele frequency [MAF] < 0.01) variants that were shared by the affected individuals and were not present in the unaffected individuals were studied further. These variants were functionally annotated using wANNOVAR to identify variants estimated to be pathogenic by SIFT, Polyphen‐2, and Mutation Taster. Variants were validated using capillary sequencing with ABI3500xL Genetic Analyzer (Applied Biosystems, Carlsbad, CA, USA) from all family members to find alleles co‐segregating with the phenotype.

### Expression of the ZNF528 in vitro

#### ZNF528 pcDNA3.1 constructs

To study the effect of the *ZNF528* c. 1282C > T/p.Arg428* variant, two expression constructs were made: a wild‐type ZFF528 (V5‐ZNF528‐WT) and a construct with the identified variant (V5‐ZNF528‐c.1282C > T). Before qPCR and Western blot experiments, cells were transiently transfected with V5‐ZNF528‐WT or V5‐ZNF528‐c.1282C>T constructs.

#### Real‐time quantitative polymerase chain reaction (qPCR)

Real‐time qPCR was carried out in duplicate using iTaq SYBR Green Supermix kit (Bio‐Rad, Hercules, CA, USA) in accordance with the manufacturer's instructions in a CFX96 Real‐Time System instrument (Bio‐Rad). Data were analyzed using the 2(−Delta Delta C(T)) –method.^(^ ^33^ ^)^ Beta‐actin (*ACTB*) and beta‐2‐microtubulin (*B2M*) were used as reference genes for HEK293 cells and hypoxanthine‐guanine phosphoribosyltransferase 1 (*HPRT1*) and succinate dehydrogenase complex flavoprotein subunit A (*SDHA*) for primary skin fibroblasts.

#### Western blot

Proteins were separated by 10% SDS‐PAGE and transferred onto 0.45‐μm PVDF membrane (Immobilon‐P, Millipore, Bedford, MA, USA). The membrane was exposed to antibodies (1:1000 dilution) V5‐HRP (Invitrogen, Carlsbad, CA, USA) or β‐actin‐HRP (Abcam, Cambridge, UK), and signal was imaged using a LAS‐3000 Luminescent Image Analyzer (FujiFilm, Tokyo, Japan). The protein bands were normalized to β‐actin and Image J software (National Institute of Mental Health, Bethesda, MD, USA) was used to quantify immunoblots.

### Protein expression and subcellular localization of ZNF528

#### Lentiviral constructs, lentivirus production, and infection of Saos‐2 cell line

Saos‐2 cell lines stably expressing V5‐ZNF528‐WT and V5‐ZNF528‐c.1282C > T were created using lentivirus infection as described in the Supplemental Material and Methods. The V5‐ZNF528‐WT and V5‐ZNF528‐c.1282 C > T expressing lentiviruses were produced in HEK293T cells using the second‐generation lentivirus packaging system. The Saos‐2 cell lines were sorted by FACS using BD FACSAria flow cytometer (BD Biosciences, Heidelberg, Germany).

#### Immunofluorescence

Saos‐2 cells overexpressing V5‐ZNF528‐WT, V5‐ZNF528‐c.1282C > T, or empty vector were fixed in 4% paraformaldehyde and permeabilized with 0.1% Triton X‐100. The cells were incubated with primary V5 antibody (Invitrogen, Carlsbad, CA, USA) and fluorescent‐conjugated secondary antibody Alexa488 (Invitrogen). To stain nucleus, the cells were incubated with 2‐(4‐amidinophenyl)‐1H‐indole‐6‐carboxamidine (DAPI) (Sigma‐Aldrich, St. Louis, MO, USA) and with TRITC‐Phalloidin (Sigma‐Aldrich) to stain actin filaments. A Zeiss LSM 780 confocal microscope was used for confocal laser scanning images analysis, using a × 40 Plan‐Apochromat objective, and analyzed by the ZEN 2011 software (Zeiss, Thornwood, NY, USA).

#### Cell viability and cytotoxicity assays

To assess whether V5‐ZNF528‐WT or V5‐ZNF528‐c.1282C > T induce cell death, cell viability and cytotoxicity assays were performed. Experiments were performed in triplicate and statistical significance was calculated by two‐tailed Student's *t* test with equal variances.

### ZNF528 in databases

Expression Atlas (www.ebi.ac.uk/gxa), the Genotype‐Tissue Expression (GTEx) v5 (www.gtexportal.org), and Cell Line Navigator (www.medicalgenomics.org/celllinenavigator) databases were used to study the expression of ZNF528 in tissues and cell lines.

### Chromatin immunoprecipitation (ChIP) sequencing

Chromatin DNA was purified by MinElute PCR Purification Kit (Qiagen, Chatsworth, CA, USA). The ChIP sequencing library was built by using the TruSeq ChIP Sample Preparation kit (Illumina). The library was sequenced using a NextSeq550 sequencing system (Illumina).

### ChIP sequencing data analysis

The peak regions across the genome (hg19) were annotated and peak‐associated genes and differentially expressed genes from RNA sequencing for V5‐ZNF528‐WT, V5‐ZNF528‐c.1282C > T were intersected. Functional enrichment analysis was performed using HOMER and Genomic Regions Enrichment of Annotations Tool (GREAT). The analysis of distribution of ChIP sequencing peaks across the genome was performed using R package ChIPseeker.

In addition, re‐analysis was performed for the ChIP sequencing data of wild‐type ZNF528 that is publicly available (http://zifrc. ccbr.utoronto.ca/) and reported earlier.^(^ ^34^ ^)^ To investigate whether the ChIP sequencing peaks are linked to genes with any functional annotations, ontology analysis was performed using GREAT. An enrichment analysis was carried out for gene ontology biological process (GO BP) terms and mouse and human phenotypes. Among these pathways, genes that had the ZNF528 binding site and had a previously known connection to bone phenotypes were identified. These genes were considered as potential ZNF528 target genes for further analyses.

### RNA sequencing

RNA was prepared from Saos‐2 cells overexpressing either V5‐ZNF528‐WT or V5‐ZNF528‐c.1282C > T and control cells expressing empty vector each with two replicates. Illumina TruSeq Stranded mRNA Library preparation kit was used according to the manufacturer's instructions. Illumina NextSeq550 platform was used to sequence the RNA libraries, with single‐ended and 76 cycle mode. The FASTQ data were prepared within BaseSpace (Illumina). Correlation of expression between replicates is presented in Supplemental Fig. S3.

### Differential gene expression analyses

#### RNA sequencing

RNA sequencing data analysis was performed as previously described^(^ ^35^ ^)^ with minor modifications. DESeq2 version 1.22.2 was applied to perform differential gene expression analysis in R (version 3.5.2). For calling differentially expressed genes, the false discovery rate (FDR) cut‐off was set to <0.01. The read counts were normalized with shifted logarithm transformation for plotting heat maps using R package pheatmap (version 1.0.12). Functional enrichment analysis using WikiPathways database was performed via HOMER. OsteoporosAtlas database was used to identify osteoporosis‐related genes, and statistical enrichment was evaluated using Pearson's chi‐square test.

#### Differentally expressed genes in patient fibroblasts

Genes identified as potential target genes in the ChIP sequencing data sets were studied in the in vitro RNA sequencing data and in the patients' primary skin fibroblasts using qPCR. Primers used in qPCR are presented in Supplemental Table S2.

## Results

### Whole‐exome data analysis

In the three affected family members, we observed altogether 239 exonic variants that were not detected in the other two unaffected individuals or in an in‐house exome data set (*n* = 71). Functional annotation identified nine rare variants with six indicating deleterious (harmful) effects through in silico prediction and three lacking such prediction. These nine variants were genotyped in all eight family members. Two single nucleotide variants (SNVs) co‐segregated with the disease: a nonsense mutation (rs150257846) in the zinc finger protein 528 gene (*ZNF528*) and a missense SNV (rs192842795) in the synapse defective 1, Rho GTPase, homolog 2 (*C. elegans*) gene (*SYDE2*) (Table 1). The SYDE2 protein is by similarity predicted to be a GTPase activator for Rho‐type GTPases (UniProtKB/Swiss‐Prot: Q5VT97). The p.Ala254Thr (rs192842795) variant lies in the region that has no homology with any other protein, shows poor sequence conservation among mammals, and was estimated to be pathogenic only by SIFT, but with low confidence because of low diversity of sequences in the database. We thus excluded this variant from further studies.

**Table 1:** Variants Co‐segregating With the Phenotype

Gene	Rs‐number	Variant^a^	Function	gnomAD Finnish	ExAc Finnish	SIFT	PP‐2	MT
*ZNF528*	rs150257846	c.1282C > T, p.Arg428*	Nonsense	0.004	0.006	D	NA	NA
*SYDE2*	rs192842795	c.760G > A, p.Ala254Thr	Missense	0.002	0.002	D^b^	B	P

gnomAD Finnish = the Genome Aggregation Database Finnish data; ExAc Finnish = Exome Aggregation Consortium Finnish data; PP‐2 = PolyPhen‐2; MT = Mutation Taster; D = deleterious/damaging; B = benign; P = polymorphism; NA = not available.

^a^ Variant location based on NM_032184 for *SYDE* and NM_032423 for *ZNF528*.

^b^ Low confidence.

**Fig 1:**
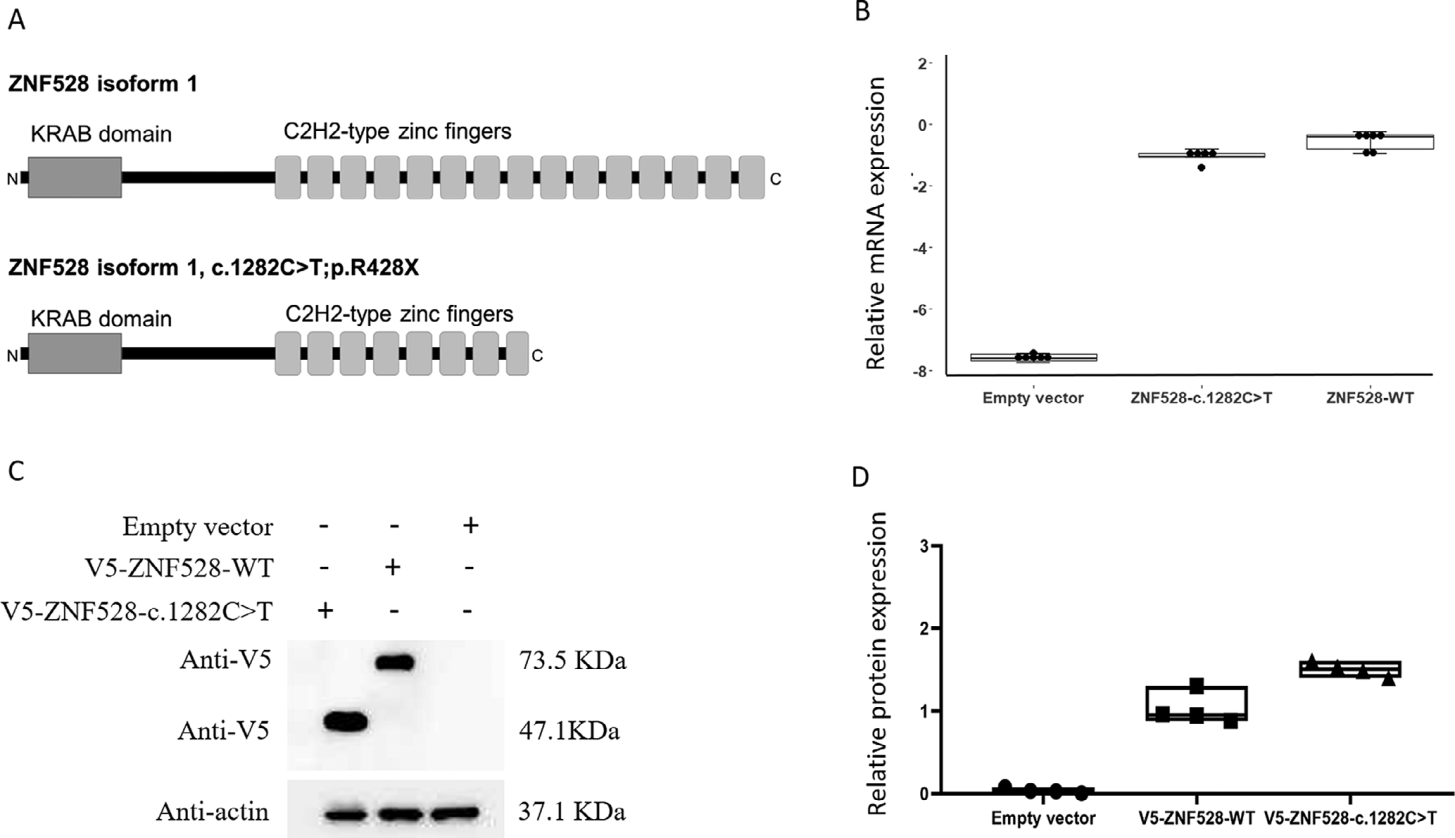
mRNA and protein expression of V5‐ZNF528‐WT and V5‐ZNF528‐c.1282C>T in HEK293 cells. (A) A schematic representation of the predicted effect of V5‐ZNF528‐c.1282C>T on ZNF528 protein structure (B) mRNA expression levels determined by qPCR analysis (six replicates) (C) Protein expression levels determined by western blot. (D) Quantification of western blot results (four technical replicates).

ZNF528 is by similarity predicted to be a zinc finger protein implicated in transcriptional regulation (UniProtKB/Swiss‐Prot: Q3MIS6). ZNF528 consists of 3994 bp organized into seven exons. The c.1282C > T/p.Arg428* (rs150257846) variant is located in the last exon. A schematic representation of the predicted effect on protein structure is presented in Fig. 1*A*. The p.Arg428* (rs150257846) variant disrupts the 8th C2H2‐type zinc finger of 15 found in the protein and will most likely have an effect on the function of ZNF528.

ZNF528 has two isoforms. Isoform 1 is 628 amino acids long and includes one krüppel associated box (KRAB) domain and 15 C2H2‐type zinc finger domains. Isoform 2 is missing the first 233 amino acids including the KRAB domain. KRAB domains typically participate in protein–protein interactions and zinc finger domains bind specific DNA motifs.^(^ ^36^ ^)^ Based on the protein structure, ZNF528 isoform 1 may function as a classical C2H2 type zinc finger transcription factor. Isoform 1 is expressed in fibroblasts based on the GTEx database (GTEx Consortium, 2013). The function of C2H2 zinc fingers lacking the KRAB domain, such as in the ZNF528 isoform 2, is unknown. Although the variant might affect both of the isoforms, we focused on ZNF528 isoform 1 because of its probable function as a DNA‐binding transcription factor and existing data on its expression.

### Expression of the ZNF528 and its variant in vitro

Western blot and qPCR were carried out to investigate if a truncated form of ZNF528 with c.1282C > T/p.Arg428* is produced. mRNA expression was observed in HEK293 cells transfected with either V5‐ZNF528‐WT or V5‐ZNF528‐c.1282C > T, with the expression of mRNA carrying the variant being lower than the wild‐type mRNA (*p* = 0.011, Fig. 1*B*). Western blotting showed protein products of sizes 73.5 and 47.1 kDa corresponding to the expected sizes of wild‐type ZNF528 and truncated ZNF528, respectively, confirming that the truncated form is produced (Fig. 1*C*, *D*; Supplemental Figs. S4 and S5).

### Subcellular localization of ZNF528 and its variant

**Fig 2:**
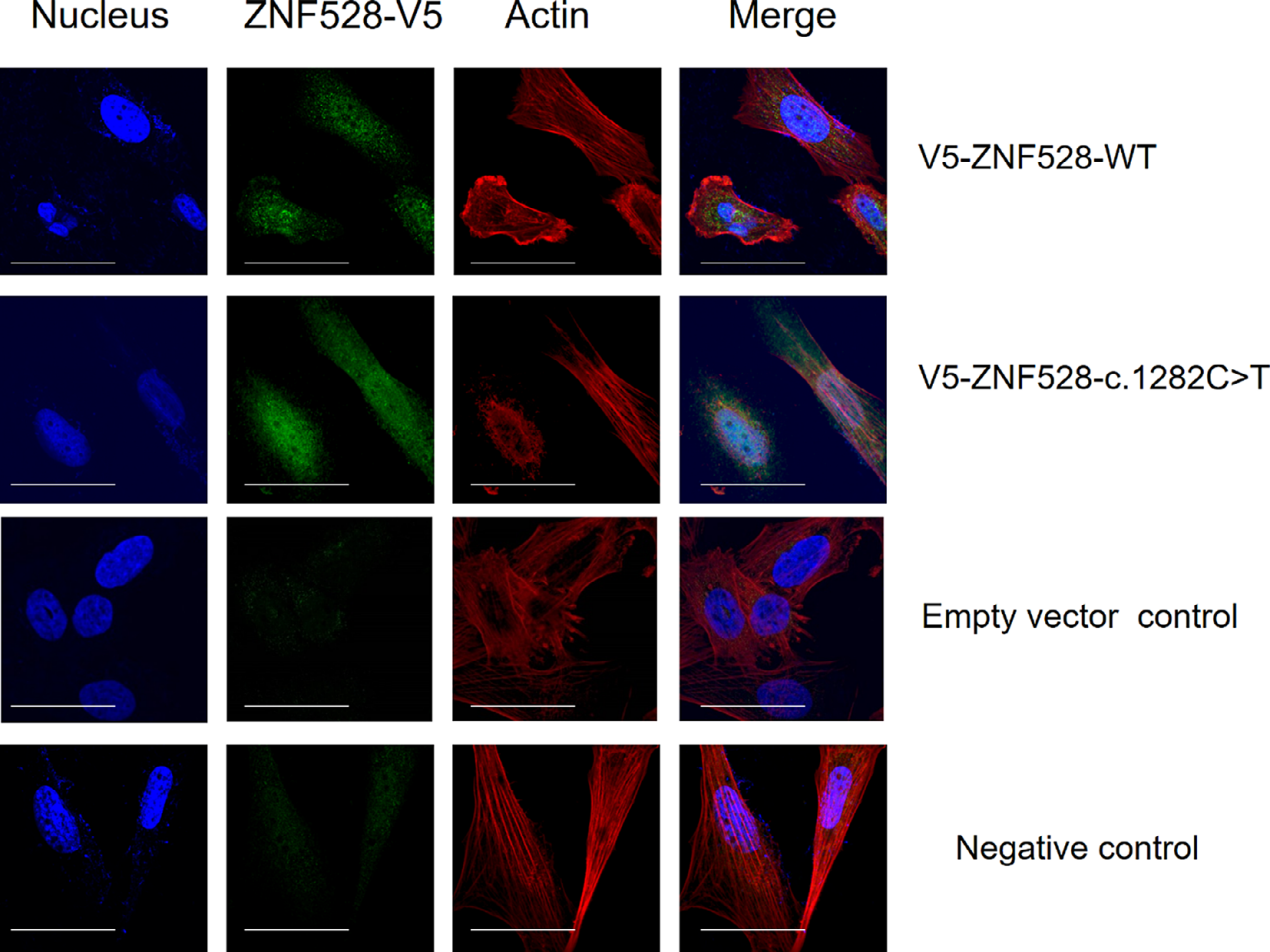
Localization of V5‐ZNF528‐WT and V5‐ZNF528‐c.1282C > T expression in Saos‐2 cells. The locations of ZNF528 (green), nucleus (blue), and actin (red) were observed through confocal imaging. The nucleus was stained with DAPI and actin filaments with TRITC‐Phalloidin. Scale bar = 100 μm.

Wild‐type ZNF528 has previously been shown to localize in the nucleus and to function as a zinc finger (Zf) transcription factor in HEK293 cell line.^(^ ^34^ ^)^ We thus performed immunofluorescence staining in the human sarcoma osteogenic (Saos‐2) cell line and observed nuclear localization of both the full‐length and truncated V5‐tagged ZNF528 proteins (Fig. 2). This was further confirmed in HEK293 cells by Western blot performed separately from protein samples of the cytosolic and nuclear fractions (Supplemental Figs. S6–S8). Overall, these results confirm that the c.1282C > T/p.Arg428* variant leads to production of the truncated ZNF528 and that it localizes in the cell nucleus, although a minor amount was also present in the cytosol. No difference was observed between ZNF528‐WT, ZNF528‐c.1282C > T and the control group in cell viability or cytotoxicity assays (Supplemental Figs. S9 and S10).

### Expression of ZNF528 in databases

*ZNF528* is expressed widely in different human tissues and cell lines. It has been detected in 53 tissues tested in the GTEx database. mRNA expression is highest in the cerebellum, thyroid, prostate, and ovary, but it is also present in bone and connective tissue cell lines (Supplemental Table S3).

### Identifying ZNF528 target pathways and genes

**Fig 3:**
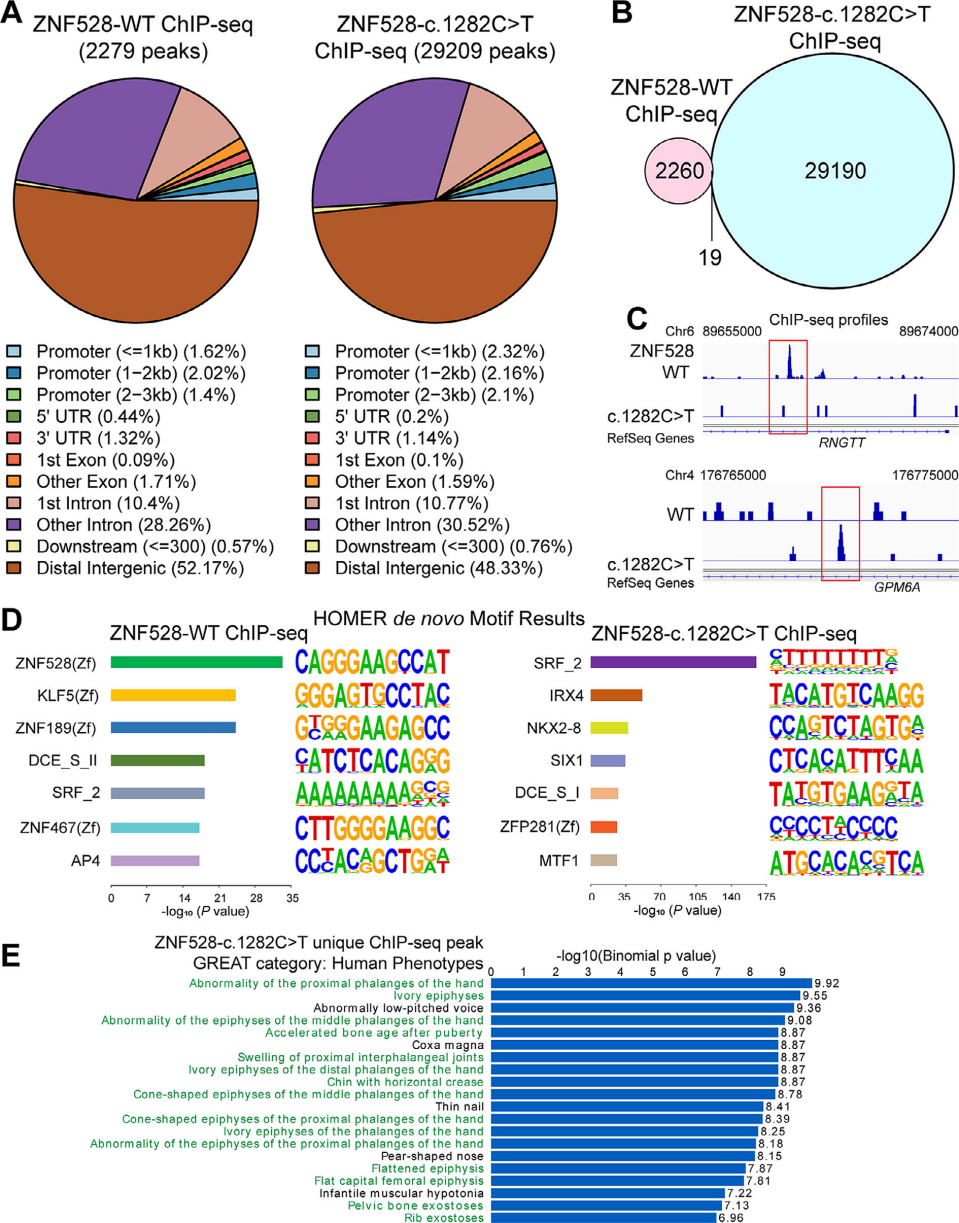
Genomewide chromatin binding of ZNF528‐WT and ZNF528‐c.1282C > T in Saos‐2 cells. (*A*) Pie charts demonstrating genomic distribution features of ZNF528‐WT or ZNF528‐c.1282C > T‐binding peaks in Saos‐2 cells. The indicated genomic features contain promoters; gene body (5′ UTR, 3′ UTR, exons, and introns); downstream elements, and distal intergenic regions. UTR = untranslated region. (*B*) The Venn diagram of overlapping peaks bound by ZNF528‐WT and ZNF528‐c.1282C > T in Saos‐2 cells. (*C*) The binding of ZNF528‐WT and ZNF528‐c.1282C > T on the representative distinct target genes, *RNGTT* and *GPM6A*. The chromosome number and position of the peaks bound by either of the proteins are indicated. (*D*) The top enriched motifs in the ZNF528‐WT or ZNF528‐c.1282C > T‐binding peaks determined by HOMER. (*E*) Functional annotation of ZNF528‐c.1282C > T peaks was performed using GREAT. Note that human phenotypes' ontology category contains data of human genotype–phenotype associations, and the *x* axis values (in logarithmic scale) correspond to the binomial raw *p* values. Bone‐related pathways are highlighted in green.

Given that both wild‐type ZNF528 and its variant localize in the nucleus, we applied ChIP sequencing to identify their genomewide binding sites and to investigate whether they suggest any potential difference in the target gene regulation. We used mouse monoclonal V5 antibody to V5‐tagged ZNF528‐WT and ZNF528‐c.1282C > T, respectively, ectopically expressed in Saos‐2 cells in parallel ChIP sequencing assays. We identified 2279 peaks for ZNF528‐WT. In contrast, we revealed more peaks (29,209) for ZNF528‐c.1282C > T (Fig. 3*A*). Only 19 binding sites were shared between ZNF528‐WT and ZNF528‐c.1282C > T (Fig. 3*B*), suggesting a distinct global shift in the genomic binding profile. Examples of ChIP‐seq profiles of the differential peak location for ZNF528‐WT and ZNF528‐c.1282C > T on two genes are shown in Fig. 3*C*. Remarkably, de novo motif analysis of ChIP sequencing peaks showed that ZNF528‐WT and ZNF528‐c.1282C > T had distinct binding motifs (Fig. 3*D*), with the greatest enrichment for ZNF528 DNA‐binding motif in ZNF528‐WT peaks, indicating functionality of the ZNF528 protein. Moreover, the ZNF528‐WT ChIP‐seq peaks are also enriched for three other types of Zf‐like motifs (KLF5, ZNF189, and ZNP467). In contrast, only one type of Zf DNA‐binding motif among top seven was found to be enriched in the ChIP sequencing peaks of ZNF528‐c.1282C > T (ZFP281), suggesting that the mutation in ZNF528 changes not only its genomewide chromatin binding profile but also DNA‐recognition properties.

ZNF528‐WT peaks showed association with genes connected with bone morphology (Fig. 3*E*; Supplemental Fig. S11*A*). Remarkably, we observed highly frequent enrichment of bone‐relevant pathways for ZNF528‐c.1282C > T peaks in GREAT categories of both human and mouse phenotypes (Fig. 3*E*; Supplemental Fig. S11*B*), presumably due to functional gain of ZNF528 caused by the mutation. In addition enrichment analysis of publicly available wild‐type ZNF528 ChIP sequencing data^(^ ^34^ ^)^ revealed several enriched GO BP, mouse phenotype, and human phenotype terms in all motif data sets (Supplemental Table S4). Many of these terms were related to musculoskeletal phenotypes and related biological processes. Especially those related to bone morphology were found enriched in the data sets (Supplemental Table S4), consistent with our ChIP sequencing data‐based analysis shown in Fig. 3*E*.

**Fig 4:**
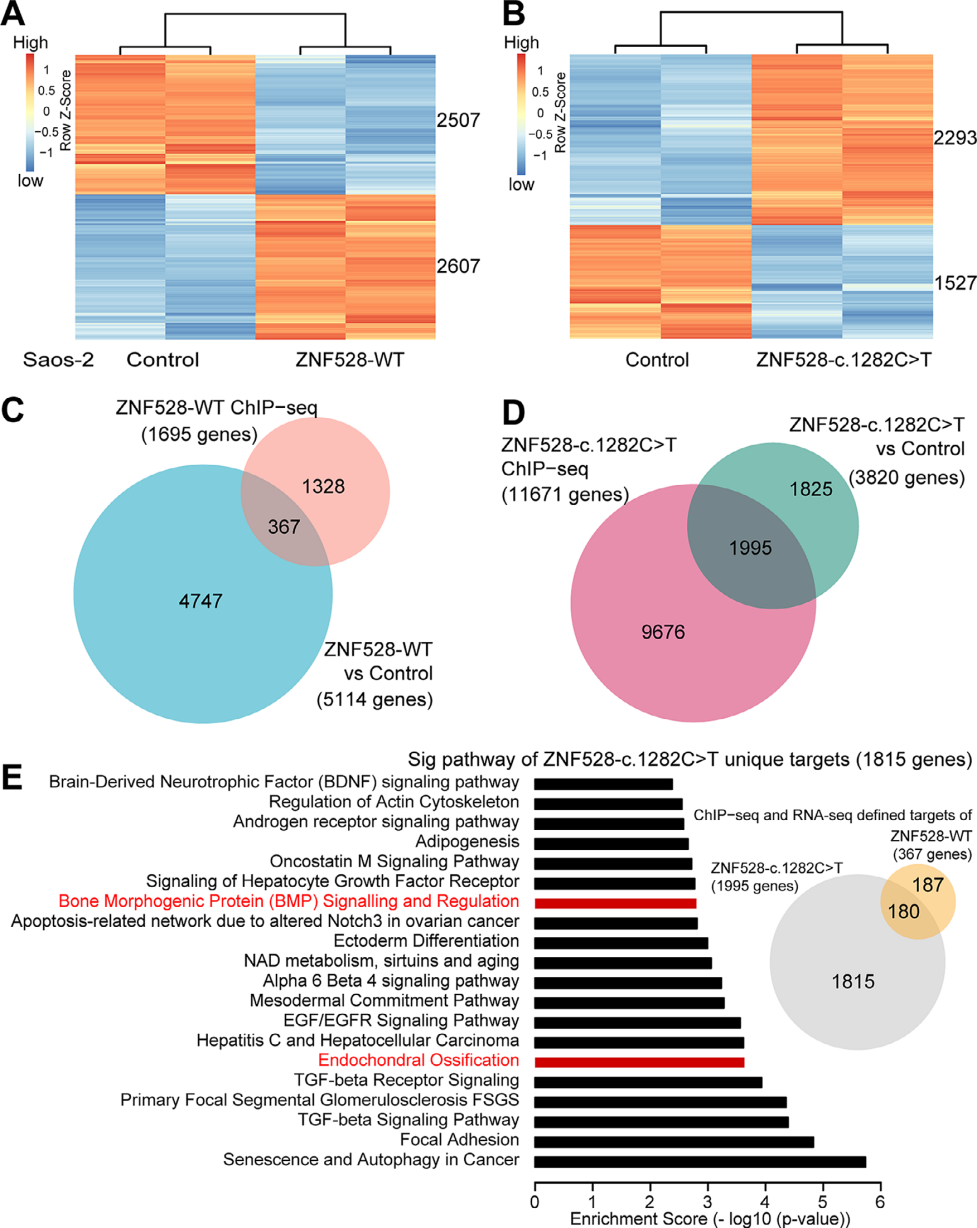
Genomewide analysis of ZNF528‐WT and ZNF528‐c.1282C > T functional target genes. (*A*, *B*) Heat maps for the expression level of genes either down‐ or upregulated by ZNF528‐WT (*A*) or ZNF528‐c.1282C > T (*B*) overexpression in Saos‐2 cells. The indicated number of genes revealed by RNA‐seq (DESeq2, FDR < 0.01). (*C*, *D*) Overlap of genes bound (ChIP‐seq) or functionally regulated (RNA‐seq) by (*C*) ZNF528‐WT or (*D*) ZNF528‐c.1282C > T. (*E*) Wikipathways enrichment analysis for ZNF528‐c.1282C > T targeted unique genes. The *x* axis presents logarithmic *p* value. Bone‐related pathways are highlighted in red.

To further identify functional target genes of ZNF528‐WT or ZNF528‐c.1282C > T and to investigate whether the difference in their genomic binding profiles is also reflected in gene expression patterns, we performed RNA sequencing analysis. Compared with a control, we found that 2607 genes were upregulated, whereas 2507 genes were downregulated by ectopic ZNF528‐WT expression (DESeq2, FDR < 0.01; Fig. 4*A*), and 2293 genes were upregulated and 1527 genes were downregulated by ectopic ZNF528‐c.1282C > T expression (DESeq2, FDR < 0.01; Fig. 4*B*). We compared the list of 1695 genes bound by ZNF528‐WT observed by Chip‐Seq to the 5114 differentially expressed genes by ectopic expression of ZNF528‐WT and found that approximately 7% (367 genes) were directly regulated by ZNF528‐WT (Fig. 4*C*). Similar analysis found that approximately 52% (1995) of expressed genes were directly targeted by ZNF528‐c.1282C > T (Fig. 4*D*). We checked the overlapping genes between the 367 ZNF528‐WT regulated genes and 1995 ZNF528‐c.1282C > T target genes and found that 187 genes were unique to ZNF528‐WT and 1815 to ZNF528‐c.1282C > T (Fig. 4*E*). Pathway enrichment analysis showed that ZNF528‐c.1282C > T unique target genes (*n* = 1815) are significantly enriched in the bone‐related pathways such as bone morphogenic protein (BMP) signalling and regulation and endochondral ossification (Fig. 4*E*). In contrast, we found no enrichment of ZNF528‐WT unique target genes (*n* = 187) in the bone‐relevant pathways (Supplemental Fig. S12*A*).

We next examined whether ZNF528 target genes are likely to be osteoporosis‐related genes by comparing the unique target genes and the OsteoporosAtlas database. Notably, the results showed that the fraction of osteoporosis‐related genes in the ZNF528‐c.1282C > T unique target gene set is significantly higher than that of ZNF528‐WT unique gene set (Supplemental Fig. S12*B*, *C*; Supplemental Table S5). These include the genes such as SOX6 and the BMP‐SMAD signaling pathway genes BMP2, BMP6, and SMAD6. Altogether, the pathway and osteoporosis‐related gene enrichment patterns on ZNF528‐c.1282C > T are dramatically different from the ZNF528‐WT targeted pathways, further supporting our findings of their differences in global genomic binding profiles (Fig. 3*B*).

For example, in the analysis of the publicly available data set,^(34^ ^)^ the most enriched GO‐BP pathways included *cartilage development involved in endochondral bone morphogenesis* and terms relating to cranial morphology. In addition, the terms *abnormal metatarsal and metacarpal bone morphology* and *increased vertebrae number* were enriched in the mouse phenotype database. In the human phenotype, database terms *abnormality of the pubic bones* and *aplasia/hypoplasia of the pubic bone* were enriched. Among these pathways, we identified genes that had the ZNF528 binding site and had a previously known connection to bone phenotypes (Supplemental Table S6). We considered these genes as potential ZNF528 target genes for further analyses.

### Expression of ZNF528 and its potential target genes

#### RNA sequencing

**Fig 5:**
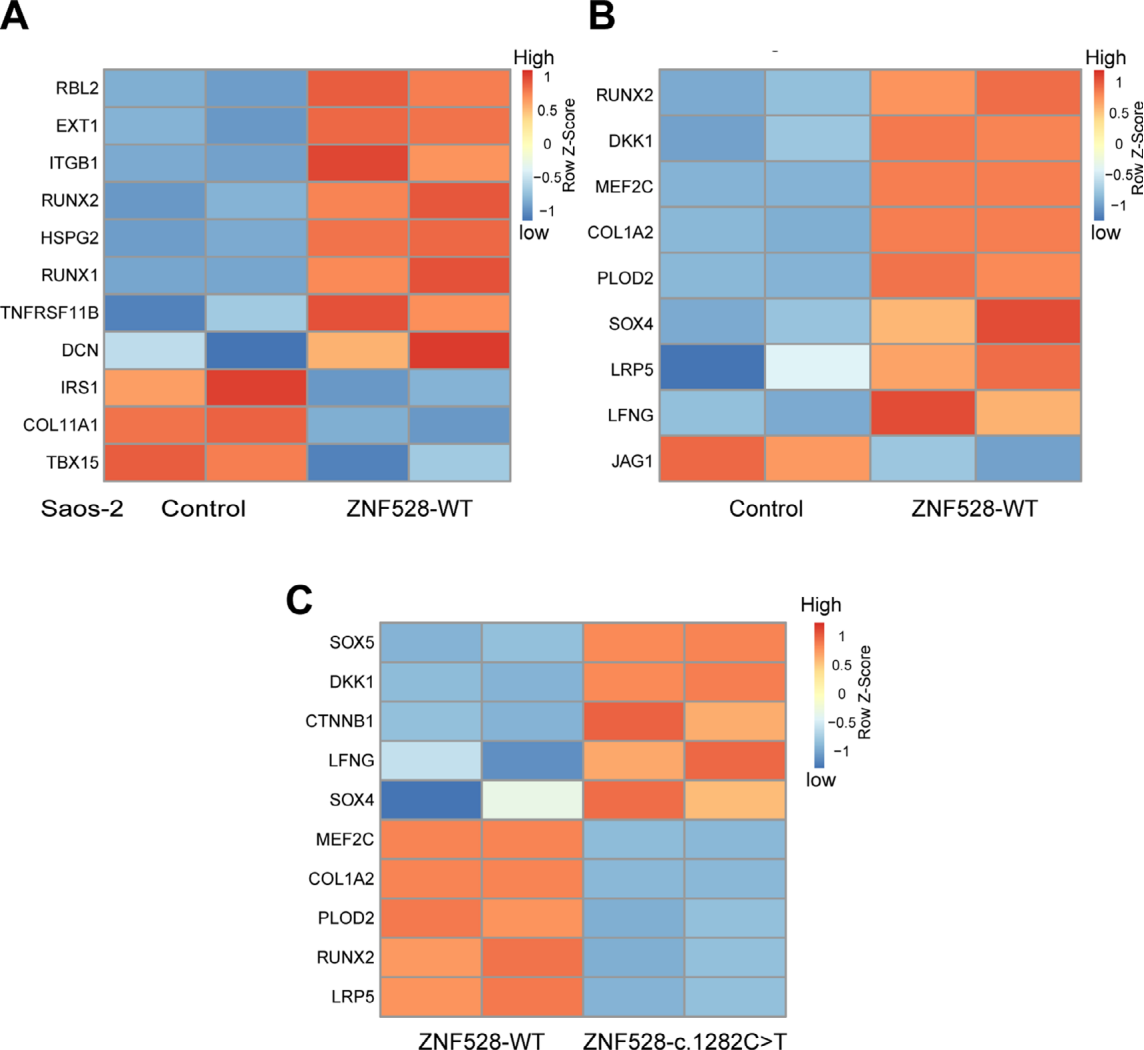
RNA sequencing analysis showing the genes that are differentially regulated by ZNF528‐WT or ZNF528‐c.1282C > T. (*A*) Significant differentially expressed genes in bone morphology relevant pathways shown in Fig. 5*E*. (*B*) Significant differentially expressed genes with ZNF528 binding motifs shown in Table 2. (*C*) Significant differentially expressed genes cross‐validate patient data shown in Table 2.

Using RNA sequencing data, we observed that eight genes were upregulated and three genes downregulated by wild‐type ZNF528 (Fig. 5*A*) in bone morphology relevant pathways (Fig. 3*E*). Furthermore, nine potential bone phenotype‐related ZNF528 target genes were differentially expressed and upregulated by ZNF528‐WT (Fig. 5*B*). We observed that 10 of the potential ZNF528 target genes presented in Supplemental Table S6 were differentially regulated by ZNF528‐c.1282C > T compared with ZNF528‐WT in our in vitro cell model (Fig. 5*C*).

#### Patient cells

Expression of ZNF528 was observed in primary skin fibroblasts. In patients carrying the c.1282C > T/p.R428X variant, the mRNA expression of ZNF528 was decreased compared with controls (Table 2). Eleven of 17 connective tissue‐related potential ZNF528 target genes were differentially expressed in the skin fibroblasts of two affected family members compared with the controls. The mRNA expression of seven genes was increased in patients compared with control: catenin beta 1 (*CTNNB1*), CYLD lysine 63 deubiquitinase (*CYLD*), Dickkopf WNT signaling pathway inhibitor 1 (*DKK1*), Estrogen receptor 1 (*ESR1*), O‐fucosylpeptide 3‐beta‐N‐acetylglucosaminyltransferase (*LFNG*), Myocyte Enhancer Factor 2C (*MEF2C*), and wntless Wnt ligand secretion mediator (*WLS*), whereas for five genes it was decreased: collagen I alpha 2 chain (*COL1A2*), Jagged 1 (*JAG1*), procollagen‐lysine,2‐oxoglutarate 5‐dioxygenase 2 (*PLOD2*), and SRY‐box 5 (*SOX5*) (Table 2). No difference was found in the patients' fibroblasts in the mRNA expression of the LDL receptor‐related protein 5 (*LRP5*), TNF superfamily member 11 (*RANKL*), R‐spondin 3 (*RSPO3*), runt‐related transcription factor 2 (*RUNX2*), SRY‐box 4 (*SOX4*), or SRY‐box 9 (*SOX9*), although LRP5 and RUNX2 were downregulated and SOX4 upregulated by ZNF528‐c.1282C > T in our in vitro analysis (Fig. 5*C*).

**Table 2:** Expression of ZNF528 and Its Potential Target Genes in Patient Fibroblasts Compared With Controls

Gene	*p* Value	Adjusted *p* value	Fold change
*ZNF528*	**0.019**	**0.0342**	**0.31↓**
Potential target genes			
*COL1A2* ^a^	**0.006**	**0.018**	**0.45↓**
*CTNNB1*	**1.174 × 10** ^**‐5**^	**0.0002**	**3.35↑**
*CYLD*	**0.007**	**0.018**	**3.04↑**
*DKK1*	**0.025**	**0.0375**	**1.67↑**
*ESR1*	**0.001**	**0.009**	**3.95↑**
*JAG1*	**0.002**	**0.009**	**0.57↓**
*LFNG*	**0.002**	**0.009**	**6.19↑**
*LRP5*	0.198	0.255	1.2
*MEF2C*	**0.008**	**0.018**	**1.73↑**
*PLOD2*	**0.025**	**0.0375**	**0.56↓**
*RANKL*	**0.048**	0.066	0.28
*RSPO3*	0.267	0.300	2.1
*RUNX2*	0.626	0.663	0.9
*SOX4*	0.868	0.868	1.05
*SOX5*	**0.018**	**0.034**	**0.27↓**
*SOX9*	0.223	0.268	1.67
*WLS*	**0.004**	**0.014**	**2.7↑**

Statistically significant values are bolded. Decreased expression in primary skin fibroblasts from patients marked with downward pointing arrow and increased expression with upward pointing arrow. Benjamini and Hochberg's false discovery rate correction was applied for the adjusted *p* values.

^a^ Patients carry heterozygous p.Arg8Phefs^*^14 deletion.

Five genes, *COL1A2*, *CTNNB1*, *DKK1*, *LFNG*, and *PLOD2*, were differentially expressed with similar trend (increased or decreased) in both the patients' fibroblasts and in the RNA sequencing experiment (Table 2; Fig. 5*C*).

## Discussion

We have identified a rare nonsense variant (c.1282C > T/p.Arg428*) in *ZNF528* in patients who were previously reported to have a deletion in *COL1A2* and suffered from severe early onset primary osteoporosis but lacked the typical features of either OI or EDS.^(^ ^32^ ^)^ The biological function of ZNF528 is poorly understood and it has not previously been associated with bone or connective tissue‐related phenotypes. The observed variant results in a truncated ZNF528 protein that locates in the cell nucleus as per the wild‐type protein. We show here that the variant leads to altered DNA binding of ZNF528 and a global shift in genomic binding profile and pathway enrichment. Furthermore, the expression of several ZNF528 target genes associated with bone‐related processes is altered in patients' cells as well as in in vitro cell model.

ZNF528 belongs to the family of KRAB C2H2 zinc finger proteins, which is the largest family of human transcription factors.^(^ ^37^ ^)^ These proteins are typically transcriptional repressors participating in regulation of embryonic development, apoptosis, neoplastic transformation, cell cycle regulation, as well as cell differentiation and proliferation.^(^ ^38^ ^)^ They bind to DNA with their zinc finger domains and the KRAB domain forms a complex with the KRAB‐associated protein 1 (KAP1) to recruit further co‐regulators.^(^ ^36^, ^39^ ^)^ In addition to DNA binding, zinc finger domains also participate in protein–protein interactions.^(^ ^40^ ^)^ Thus, the loss of zinc finger domains may also alter the interaction of ZNF528 with other proteins.

ZNF528 is likely to have multiple biological roles, as supported by the wide expression pattern observed in the GTEx database. This was further supported by our results of the enrichment analyses of ChIP sequencing data sets, where in addition to the bone‐related pathways, terms in other biological processes were enriched. In general, zinc finger family members tend to be widely expressed and participate in multiple different biological pathways.^(^ ^36^, ^38^ ^)^ For example, ZNF521 and ZNF423 are widely expressed and in addition to skeletal pathways they have been described to have a role in mitochondrial fatty acid synthesis, cancer, and Alzheimer's disease.^(^ ^41^, ^42^, ^43^ ^)^ Our data suggest that ZNF528 could be a regulator of bone‐related biological functions as functional annotation of ZNF528‐WT peaks showed enrichment in bone‐related mouse phenotype ontology category and similar results were obtained from analysis of ChIP data from HEK293 cells.

The variant c.1282C > T/p.Arg428* altered the ZNF528 DNA‐binding motif and led to reprogramming of the global genomic binding (Fig. 5). This finding was supported by the fact that we did not observe enrichment of GREAT GO categories among the ZNF528‐c.1282C > T peak‐linked genes. As the DNA‐recognition properties of ZNF528 are altered, the target genes and pathways for ZNF528‐WT and ZNF528‐c.1282C > T are very distinct from each other, suggesting that in addition to losing the original function, there may be an acquisition of novel functions via alterations of the DNA‐binding motif.

RNA sequencing showed that wild‐type ZNF528 directly regulated 367 genes, many of which belong to cell cycle‐ and cancer‐related pathways (Fig. 4; Supplemental Fig. S12*A*). This is not completely unexpected as the used cell line, Saos‐2, is an osteosarcoma cell line. However, our results show that the c.1282C > T/p.Arg428* variant does alter the underlying biology and function of ZNF528. More detailed examination of RNA sequencing results identified several bone morphology‐related genes likely regulated by both wild‐type and mutated ZNF528 (Fig. 4; Supplemental Fig. S12*A*). More osteoporosis‐related genes were affected by ZNF528‐c.1282C > T compared with ZNF528‐WT. Thus, the variant may enhance the binding of ZNF528 in bone‐related target genes supporting the role of this variant in osteoporosis pathogenesis (Supplemental Fig. S12*B*, *C*).

To investigate the effect of the c.1282C > T/p.Arg428* variant on the regulation of bone‐associated genes, 17 potential ZNF528 target genes were studied in primary skin fibroblasts obtained from patients. Eleven of 17 potential ZNF528 target genes were differentially expressed in patients' cells compared with controls. Five of these, including *COL1A2*, were also differentially expressed in the RNA sequencing data with similar trend (increased or decreased expression) when comparing ZNF528‐WT and ZNF528‐c.1282C > T. These five genes, *COL1A2*, *CTNNB1*, *DKK1*, *LFNG*, and *PLOD*, are promising targets for ZNF528 regulation in bone and their expression may be altered by the c.1282C > T/p.Arg428* variant.

*CTNNB1* and *DKK1* are part of WNT signaling pathway. Studies with mice have shown that β‐catenin is necessary for controlling bone development and homeostasis.^(^ ^44^, ^45^ ^)^ Stabilization of β‐catenin in mature osteoblasts leads to high bone mass phenotype, whereas lack of β‐catenin leads to osteopenia in mice.^(^ ^46^ ^)^ DKK1 is an inhibitor of WNT signaling and variants in *DKK1* have been associated with juvenile osteoporosis.^(^ ^47^ ^)^ *CTNNB1* and *DKK1* were upregulated by c.1282C > T/p.Arg428* compared with ZNF528‐WT, suggesting that the variant might contribute to the patients' phenotype via this pathway.

*LFNG* was similarly upregulated by the c.1282C > T/p.Arg428* variant. LFNG participates in the Notch signaling pathway by inhibiting the Notch ligand JAG1,^(^ ^48^ ^)^ which is required for normal trabecular bone formation and is the most highly expressed Notch ligand during skeletal development.^(^ ^49^, ^50^ ^)^ LFNG is a mediator of somite segmentation and patterning during embryogenesis and modulates Notch signaling by decreasing the binding of JAG1 to NOTCH2.^(^ ^51^ ^)^ Mutations in LFNG have also been associated with spondylocostal dysostosis.^(^ ^52^ ^)^

The c.1282C > T/p.Arg428* variant had an opposite effect on PLOD2, which was downregulated in both patients' cells and in the in vitro model. It is a membrane‐bound enzyme localized in endoplasmic reticulum and catalyzes the hydroxylation of lysyl residues in collagens. Deficiency in lysyl hydroxylase activity is linked with some cases of kyphoscoliosis type of Ehlers‐Danlos syndrome.^(^ ^53^ ^)^ Mutations in *PLOD2* are associated with Bruck syndrome that is characterized by OI and congenital joint contractures.^(^ ^54^, ^55^, ^56^ ^)^

*COL1A2* also harbors the ZNF528 binding site, making it a potential target gene for ZNF528. We did observe decreased expression of *COL1A2* in patients' mRNA, but this is consistent with assumption of nonsense mediated decay caused by the p.Arg8Phefs^*^14 deletion.^(^ ^32^ ^)^ The patients did not carry pathogenic variants in any of the other potential ZNF528 target genes. It is not possible to differentiate the putative effect of ZNF528 on the *COL1A2* expression and neither can we exclude the possibility that the *COL1A2* deletion affects the expression of target genes in the patients' fibroblasts. However, the *COL1A2* expression and the expression of the five aforementioned potential target genes were altered by the c.1282C > T/p.Arg428* variant in the in vitro RNA sequencing experiment. This implies that the observed ZNF528 variant affects the expression of these genes independently from the *COL1A2* Arg8Phefs^*^14 deletion. It is not, however, possible to determine the independent contributions of the COL1A2 deletion and the ZNF528 variant to the phenotype in this family.

In addition to these genes that are likely to contribute to the patients' phenotype, our results indicated several other interesting ZNF528 target genes, but for which the effect of c.1282C > T/p.Arg428* remained unclear (Fig. 5; Table 2). These include *MEF2C* and *SOX5*, which were differentially regulated in both patient and RNA sequencing data but seemed to have an opposite effect between the data sets. MEF2C is part of the WNT signaling pathway, where it binds to sclerostin (SOST) enhancer and induces SOST expression, which further inhibits WNT signaling. Lack of SOST leads to high bone mass phenotype.^(^ ^57^ ^)^ MEF2C has also been shown to control bone development by activating chondrocyte hypertrophy.^(^ ^58^ ^)^ *SOX5* encodes a member of Sry‐related transcription factors that regulate chondrocyte differentiation and proliferation.^(^ ^59^ ^)^ SOX5 has also been associated with chronic osteochondropathy called Kashin‐Beck disease that affects joints and bones.^(^ ^60^ ^)^ Our data support the role of ZNF528 in the regulation of the gene expression of *MEF2C* and *SOX5*, but it is not clear if this has an effect on the patients' phenotype. Transcription factors such as ZNF528 have typically wide effect on the transcriptome both directly and indirectly. The function of transcription factors is also often dependent, at least partially, on cell type or is tissue specific, and pinpointing the effects of particular variant in specific phenotype is challenging. Further research is necessary to fully understand the role of ZNF528 and the observed variant in primary osteoporosis. Overall, we provide new information on the function of ZNF528 and show that it regulates genes and pathways important for bone development.

In conclusion, we identified a c.1282C > T/p.Arg428* variant in *ZNF528* in a family with severe early onset primary osteoporosis and confirmed that this variant leads to production of truncated ZNF528 in the cell nucleus. The function and targets of ZNF528 have not been previously reported, but our results indicate that ZNF528 may function as a transcriptional regulator in several bone‐related pathways. We identified five potential target genes for ZNF528 that were differentially expressed in both patients' fibroblasts compared with control cells and in the in vitro RNA sequencing experiment. The identified *ZNF528* variant may thus contribute to the bone phenotype in the investigated family. These observations suggest that the skeletal phenotype may even in monogenic disorders such as osteogenesis imperfecta be modified by other genes.

## Disclosures

All authors state that they have no conflicts of interest.

## Author contributions

**SS:** Conceptualization; formal analysis; investigation; writing‐original draft; writing‐review and editing. **JX:** Investigation; writing‐review and editing. **QZ:** Formal analysis; writing‐review and editing. **ML:** Formal analysis; writing‐review and editing. **AC:** Investigation; writing‐review and editing. **LR:** Investigation; writing‐review and editing. **OM:** Conceptualization; resources; writing‐review and editing. **GW:** Conceptualization; resources; writing‐original draft; writing‐review and editing. **MM:** Conceptualization; resources; writing‐original draft; writing‐review and editing.

## Data Availability Statement

The RNA‐seq and ChIP‐seq data sets have been deposited in the European Nucleotide Archive (ENA) under accessions PRJEB39746 and PRJEB39772.

### Peer review

The peer review history for this article is available at https://publons.com/publon/10.1002/jbmr.4145.

## Supporting information


**Appendix S1**. Supporting information
