# Supplementary material for: Exome Sequencing Reveals a Phenotype Modifying Variant in ZNF528 in Primary Osteoporosis With a COL1A2 Deletion
Source: J Bone Miner Res. 2020 Aug 26;35(12):2381–92. doi: 10.1002/jbmr.4145 (PMC7757391; doi:10.1002/jbmr.4145)
Supplement: Supplementary file 1 — Appendix S1. Supporting information [file JBMR-35-2381-s001.pdf]

Supplemental Material

Table of Contents

Supplemental methods 2
Whole exome data analysis 2
Expression of the ZNF528 in vitro 2
Protein expression and subcellular localization of ZNF528 5
Cell viability and cytotoxicity assay 6
Chromatin immunoprecipitation (ChIP) sequencing 7
ChIP sequencing data analysis 7
Differential gene expression analyses 9
Supplemental Figures 11
Supplemental Figure S1 11
Supplemental Figure S2 12
Supplemental Figure S3 13
Supplemental Figure S4 14
Supplemental Figure S5 15
Supplemental Figure S6 16
Supplemental Figure S7 17
Supplemental Figure S8 18
Supplemental Figure S9 19
Supplemental Figure S10 20
Supplemental Figure S11 21
Supplemental Figure S12 22
Supplemental Tables 23
Supplemental Table S1 23
Supplemental Table S2 24
Supplemental Table S3 25
Supplemental Table S4 25
Supplemental Table S5 31
Supplemental Table S6 36

Supplemental methods

Whole exome data analysis

DNA samples from five family members (Supplemental Figure S1) were studied using whole exome sequencing. DNA was fragmented and enriched at the BGI (http://www.genomics.cn/en/index) by using NimbleGen SeqCap EZ Human Exome v3.0 kit. The enriched DNA was subjected to Illumina HiSeq sequencing. Clean reads were then aligned with the reference genome (hg19) and quality controlled according to the BGI Bioinformatics pipeline.

Single nucleotide variants (SNV) or small insertions or deletions (ins/dels) were detected (at least 50x coverage by reads, base call with Phred quality scores greater than 20) and filtered using Chipster. Data was analyzed using BEDTools software. Only private or rare (minor allele frequency (MAF) < 0.01) variants that were shared by the affected individuals and not present in the unaffected individuals or in the in-house exome set (N=71) were studied further. These remaining variants were functionally annotated using wANNOVAR to identify variants estimated to be pathogenic by SIFT, Polyphen-2 and Mutation Taster. Variants without annotations were not excluded. Variants were validated using capillary sequencing with ABI3500xL Genetic Analyzer (Applied Biosystems) from all family members to find alleles co-segregating with the phenotype. The frequencies for co-segregating variants in the general Finnish population were obtained from the The Exome Aggregation Consortium browser (ExAC, https://exac.broadinstitute.org/) and the Genome Aggregation Database (gnomAD, https://gnomad.broadinstitute.org/).

Expression of the ZNF528 in vitro

ZNF528 pcDNA3.1 constructs. To study the effect of the *ZNF528* c. 1282C>T/p.Arg428* variant two expression constructs were made: a wild type ZFF528 (V5-ZNF528-WT) and a construct with the identified variant (V5-ZNF528-c.1282C>T). Briefly, the wild type ZNF528 cDNA was commercially acquired (GenScript, Piscataway, NJ, USA) and the c. 1282C>T/p.Arg428* variant was introduced

using QuickChange site directed mutagenesis kit (Stratagene, La Jolla, CA, USA). The cDNA was amplified using PCR, cut with XbaI and BamHI restriction enzymes and ligated into pcDNA3.1. (-) expression vector. V5-tag was introduced to the N-terminus. The constructs were amplified by transformation into XL1Blue *E.coli* strain and culturing overnight at 37°C in 100 ml of LB broth (1g/100ml NaCl, 1g/100ml Tryptone, 0.5g/100ml yeast extract) with thiamine, tetracycline and ampicillin. The constructs were extracted using QIAfilter maxi kit (Qiagen, Chatsworth, CA, USA) and verified by capillary sequencing.

Cell culture. Human embryonic kidney (HEK) 293 cells were cultured using 100 mm plates at 37°C, 5 % CO2 in DMEM containing 10 % FBS, 0.1 % penicillin (Sigma-Aldrich, St Louis, MO) and 0.01 % Fungizone (Cambrex Bio Science, Walkersville, MD). After 48 hours the cells were divided 1:3 and cultured for 24 hours and then plated on a six-well plate (3.5×10^5 cells/well). Prior to qPCR experiment cells were transiently transfected with V5-ZNF528-WT or V5-ZNF528-c.C1282T constructs using FuGENE HD transfection reagent (Promega, Madison, WI, USA). Empty vector was used as a control. Transfected cells were further cultured for 24 hours prior to the RNA extraction. Two 100 mm plates of each cell line were divided into two portions for RNA extraction creating four replicates of each sample. For western blot Lipofectamin 3000 (Invitrogen, Carlsbad, CA, USA) was used to transfect the HEK293 cells with V5-ZNF528-WT or V5-ZNF528-c.C1282T constructs. The Saos-2 osteosarcoma cells were cultured at 37°C and 5 % CO_2 in McCoy's 5a Modified Medium (ATCC, Manassas, VA, USA) containing 15 % fetal bovine serum. The cells were cultured on 100 mm plates for five days and then divided 1:2.

Culturing primary skin fibroblasts. Primary skin fibroblasts were cultured to obtain total RNA for qPCR analysis to study the expression of ZNF528 target genes in patient cells. Primary skin fibroblasts from two patients, and three age and gender matched unrelated controls were cultured at 37°C and 5 % CO_2 in Dulbecco’s modified Eagle medium (DMEM) with high glucose and GlutaMAX supplement (Gibco, Grand Island, NY, USA) containing 10 % fetal bovine serum (FBS)

(HyClone, Logan, UT, USA), non-essential amino acids (Sigma-Aldrich, St Louis, MO, USA), 5 % Sodium pyruvate (Sigma-Aldrich, St Louis, MO, USA), 0.1 % penicillin (Sigma-Aldrich, St Louis, MO, USA), 0.01 % Fungizone (Cambrex Bio Science, Walkersville, MD, USA) and 0.5 µg/ml sodium L-ascorbate (Sigma-Aldrich, St Louis, MO, USA). The primary skin fibroblasts were cultured using 100 mm CellBIND surface plates (Corning, LifeSciences, Lowell, MA, USA). After 48 hours the cells were divided 1:3 and cultured for 24 hours prior to the RNA extraction. Two 100 mm plates of each cell line were divided into two portions for RNA extraction creating four replicates of each sample.

*Real-time quantitative polymerase chain reaction (qPCR).*Total RNA was extracted from HEK293 cells and primary skin fibroblasts using E.Z.N.A Total RNA Kit (Omega Bio-Tek, Norcross, GA, USA) with RNase-free DNase (Omega Bio-Tek, Norcross, GA, USA) treatment and cDNA was synthesized using iScript cDNA Synthesis Kit (Bio-Rad, Hercules, CA). Real-time qPCR was carried out in duplicate using iTaq SYBR Green Supermix kit (Bio-Rad, Hercules, CA) in accordance with the manufacturer's instructions in a CFX96 Real-Time System instrument (Bio-Rad, Hercules, CA). Data was analyzed using the 2(-Delta Delta C(T)) –method [(39)]. Beta-actin (*ACTB*) and beta-2-microtubulin (*B2M*) were used as reference genes for HEK293 cells and hypoxanthine-guanine phosphoribosyltransferase 1 *(HPRT1)* and succinate dehydrogenase complex flavoprotein subunit A *(SDHA)* for primary skin fibroblasts.

Western blot. HEK293 cells were centrifuged in PBS buffer with 0.6M NaCl, 1 % Triton X-100 and a cocktail of protease inhibitors (Roche, Basel, Switzerland). For nuclear/cytoplasmic separation, we used the NE-PER™ Nuclear and Cytoplasmic Extraction kit (Thermo Scientific, Waltham, MA, USA). Loading buffer (Thermo Scientific, Rockford, IL, USA) was added and the proteins were denatured in 95°C for 5 minutes. Proteins were then separated by 10 % SDS-PAGE and transferred onto 0.45-µm PVDF membrane (Immobilon-P, Millipore, Bedford, MA, USA) using a Semi-Dry transfer cell system (Trans-Blot SD, Bio-Rad, Hercules, CA, USA). The membrane was blocked for

unspecific protein binding using 5 % nonfat milk in TBST (50 mM Tris-HCl, pH 7.5, 150 mM NaCl, 0.05 % Tween-20) for 30 minutes. The membrane was then exposed to antibodies (1:1000 dilution) V5-HRP (Invitrogen, Carlsbad, CA, USA) or β-actin-HRP (Abcam, Cambridge, UK). Chemiluminescence signal was imaged using a LAS-3000 Luminescent Image Analyzer (FujiFilm, Tokyo, Japan). The protein bands were normalized to β-actin and Image J software (National Institute of Mental Health, Bethesda, MD) were used to quantify immunoblots.

Protein expression and subcellular localization of ZNF528

Lentiviral constructs, lentivirus production and infection of Saos-2 cell line. Saos-2 cell lines stably expressing V5-ZNF528-WT and V5-ZNF528-c.1282C>T were created using lentivirus infection. The fragment of V5-ZNF528-c.1282C>T was inserted to the lentivirus vector pLVET-IRES-GFP, which harbors the GFP gene as an independent expression unit in its backbone. The V5-ZNF528-WT and V5-ZNF528-c.1282 C>T expressing lentiviruses were produced in HEK293T cells using the second-generation lentivirus packaging system. Briefly, 60 % – 80 % confluent HEK293T cells were co-transfected with lentiviral transfer vector (5 µg each), pMD2.G (envelope plasmid, 1.25 µg), pPAX2 (packaging plasmid 3.75 µg), using 20 µl Lipofectamine 2000 (Invitrogen, Carlsbad, CA, USA). The HEK293T cells were grown in low glucose DMEM (Invitrogen, Carlsbad, CA, USA) containing 10 % FBS, 0.1 % penicillin and streptomycin. The growth medium was replaced with fresh medium 24 hours after transfection and the virus-containing medium was collected every 12 hours up to six days. Lentiviruses were passed through 0.45 mm filter, snap frozen in liquid nitrogen and stored. For viral infection, the Saos-2 cells were seeded in 6-well plate 18 hours prior to infection and grown to 60 %–70 % confluency. The medium was replaced with lentivirus-containing medium with 8 mg/ml polybrene (Sigma-Aldrich, St Louis, MO, USA). After 24 hours of incubation the virus-containing medium was removed and replaced with fresh medium. The Saos-2 cell lines were sorted by FACS using BD FACSAria flow cytometer (BD Biosciences, Heidelberg, Germany).

Immunofluorescence. Saos-2 cells overexpressing V5-ZNF528-WT, V5-ZNF528-c.1282C>T were seeded on coverslips. Saos-2 cell line transfected with the empty vector was used as control. The cells were fixed in 4 % paraformaldehyde PBS buffer for 20 minutes, then quenched with 200 mM glycine in PBS for 20 minutes. The cells were further permeabilized with 0.1 % Triton X-100, blocking solution (0.5 % bovine serum albumin, 0.2 % fish gelatin in PBS) for 50 min at room temperature. The cells were incubated with primary V5 antibody (Invitrogen, Carlsbad, CA, USA) and blocking buffer overnight at 4°C and then rinsed three times with blocking buffer at room temperature. Samples were further incubated with fluorescent-conjugated secondary antibody Alexa488 (Invitrogen, Carlsbad, CA, USA) and blocking buffer for 1 hour and washed three times with PBS. The cells were incubated with 2-(4-amidinophenyl)-1H-indole-6-carboxamidine (DAPI) (Sigma-Aldrich, St Louis, MO, USA) to stain nucleus and with TRITC-Phalloidin (Sigma-Aldrich, St Louis, MO, USA) to stain actin filaments. The cells were washed once with PBS before being mounted with Immu-Mount (Thermo Scientific, Waltham, MA, USA). A Zeiss LSM 780 confocal microscope was used for confocal laser scanning images analysis, using a × 40 Plan-Apochromat objective, and analyzed by the ZEN 2011 software (Zeiss, Thornwood, NY, USA).

Cell viability and cytotoxicity assay

The cells of different groups were trypsinized and seeded into 96 well culture plates with 2 x 10^3 each well. Cell viability was determined by using Cell Proliferation kit II (11465015001, Roche). We collected the data at the indicated time by measuring the absorbance at 450 nm according to the manufacturer's instructions. The cytotoxicity of cells were tested by CellTox Green Cytotoxicity Assay kit (G8743, Promega). The cells from each group were resuspended as 4 x 10^3 each well. The fluorescence was read with 485nm excitation source and 520nm emission filter. Data was obtained from triplicate wells and statistical significance was calculated by the two-tailed Student's t test with equal variances.

Chromatin immunoprecipitation (ChIP) sequencing

Saos-2 cells were cross linked in formaldehyde (final concentration one percent) for 10 minutes, and 125 mM glycine was used to stop the reaction. A hypotonic lysis buffer (10 mM KCl, 10% glycerol, 20 mM Tris-Cl, pH 8.0, 2 mM DTT, and a cOmplete EDTA-free protease inhibitor cocktail (Sigma-Aldrich, St Louis, MO, USA) was used to suspend cell pellets followed by incubation at 4°C for 50 minutes. The nuclei were suspended using SDS lysis buffer (50 mM Tris-HCl, 10 mM EDTA, pH 8.1, 0.5 % SDS, cOmplete EDTA-free protease inhibitor cocktail (Sigma-Aldrich, St Louis, MO, USA)). The chromatin was fragmented to an average size of 300 bp using a Q800R sonicator (Q Sonica, LLC., Newtown, USA). 70 µl of Dynabead protein G (Invitrogen, Carlsbad, CA, USA) was added to each sample. The samples were washed twice with blocking buffer (0.5 % BSA, 150 mM NaCl, 20 mM Tris-HCl, pH 8.0, 2 mM EDTA, 1 % Triton X-100, and , cOmplete EDTA-free protease inhibitor cocktail (Sigma-Aldrich, St Louis, MO, USA)) and then incubated with 7 µg of antibodies against the target proteins or control IgG in 1000 ml blocking buffer for 10 hours. The chromatin lysate (250 mg) was mixed with bead/antibody complexes and incubated at 4°C for 12 hours. The complex of antibodies/beads and chromatin was washed 10 times with RIPA buffer (50 mM Tris-HCl, pH 7.6, 150 mM NaCl, 1 mM EDTA, 0.1 % SDS, 1 % NP40, 0.5 % sodium deoxycholate). DNA-protein complexes were separated from beads by extraction buffer (10 mM Tris-HCl, pH 8.0, 1 mM EDTA, and 1 % SDS) and Proteinase K and NaCl were added followed by incubation overnight at 65°C. DNA was purified by MinElute PCR Purification Kit (Qiagen, Chatsworth, CA, USA). The ChIP sequencing library was built by using the TruSeq ChIP Sample Preparation kit (Illumina, San Diego, CA, USA). The library was sequenced using a NextSeq550 sequencing system (Illumina, San Diego, CA, USA).

ChIP sequencing data analysis

Motif analysis and peak annotation. FastQC was used to assess the quality of raw sequence reads and Trimmomatic version 0.35 was used to remove adaptors and trim reads. Processed reads were next

aligned to the human genome (hg19) using BWA mapper. The creation of tag directories, peak calling and motif analysis were performed using HOMER. The peak regions across the genome hg19 were annotated and peak-associated genes and differentially expressed genes from RNA sequencing for V5-ZNF528-WT, V5-ZNF528-c.1282C>T were intersected.

Gene-Ontology enrichment analyses. Functional enrichment analysis was performed using HOMER. The analysis of distribution of ChIP sequencing peaks across the genome was performed using R package "ChIPseeker" version v1.18.0.

A total of 2260 and 29190 unique peaks for the V5-ZNF528-WT and V5-ZNF528-c.1282C>T respectively, were obtained after excluding 19 common binding sites. Genomic Regions Enrichment of Annotations Tool (GREAT) was employed to predict functions of cis-regulatory regions for ZNF528-WT ChIP-Seq and ZNF528-c.1282C>T ChIP-Seq unique binding sites. Gene regulatory domain was defined with the parameter "single nearest gene" and background regions was selected as "whole genome". For large data sets, the option "significant By Region-based Binomial" that applies a binomial test over genomic regions was further selected.

In addition, re-analysis was performed for the ChIP sequencing data of wild type ZNF528 that is publicly available (http://zifrc. ccbr.utoronto.ca/) and reported earlier [(34)]. The downloaded data included three different motif data sets: 1) the ZNF528 motifs predicted by bacterial one-hybrid recognition code (B1H-RC motifs), containing 3367 motifs; 2) the de novo motifs identified by MEME, containing 11 841 motifs and 3) the de novo motifs most similar to the B1H-RC motifs trimmed based on their alignment with the B1H-RC motifs (De Novo Similar to B1H-RC Trimmed Motifs), containing 8 009 motifs.

To investigate whether the ChIP sequencing peaks are linked to genes with any functional annotations, ontology analysis was performed using the GREAT. An enrichment analysis was carried out for Gene ontology biological process (GO BP) terms and Mouse and Human phenotypes was

performed. Amongst these pathways, genes that had the ZNF528 binding site and had a previously known connection to bone phenotypes were identified. These genes were considered as potential ZNF528 target genes for further analyses.

RNA sequencing

RNA was prepared from Saos-2 cells overexpressing either V5-ZNF528-WT or V5-ZNF528-c.1282C>T and control cells expressing empty vector each with three replicates. The quality and quantity of total RNA was measured by Agilent Bioanalyzer 2100 (Agilent Technologies, Santa Clara, CA, USA) with Eukaryote Total RNA Nano Kit (Agilent Technologies, Santa Clara, CA, USA), and Qubit RNA Broad Range kit (Life Technologies, Carlsbad, CA, USA). Illumina TruSeq Stranded mRNA Library preparation kit (Illumina, San Diego, CA, USA) was used according to the manufacturer's instructions. A Bioanalyzer 2100 was used for the assessment of quantification and quality of libraries with DNA 1000 Kit (Agilent Technologies, Santa Clara, CA, USA), qPCR KAPA Library quantification kit (Roche, Basel, Switzerland) and Qubit Broad Range DNA-kit. Illumina NextSeq550 platform was used to sequence the RNA libraries, with single-ended and 76 cycle mode. The FASTQ data was prepared within BaseSpace (Illumina, San Diego, CA, USA).

Differential gene expression analyses

DESeq2 version 1.22.2 was applied to perform differential gene expression analysis in R (version 3.5.2). For calling differentially expressed genes the false discovery rate (FDR) cutoff was set to < 0.01. The read counts were normalized with shifted logarithm transformation for plotting heatmaps using R package pheatmap (version 1.0.12).

Functional enrichment analysis using WikiPathways database was performed through HOMER software. We performed the gene overlapping analysis for genes from ZNF528 WT and ZNF528-c.1282C>T obtained from the overlap of RNA-Seq differentially expressed genes and ChIP-Seq peak annotated genes. Common overlapping genes were excluded and unique genes of ZNF528 WT and

ZNF528-c.1282C>T were then converted to Entrez Gene IDs and followed by the submission to HOMER "findGO.pl" function for the assessment of the enrichment of ontology categories. The "bg" parameter of the "findGO.pl" was set as default.

We checked the common overlapping genes between ZNF528 WT or ZNF528-c.1282C>T with osteoporosis-related genes from OsteoporosAtlas (http://biokb.ncpsb.org/osteoporosis/), which is a manually curated database for human osteoporosis-related genes. We then checked the statistical enrichment of osteoporosis-related genes in the ZNF528 WT or ZNF528-c.1282C>T unique gene lists using Pearson's Chi-squared test in R (V 3.6.2).

Supplemental Figures

Supplemental Figure S1

Pedigree of the family with primary osteoporosis. Black symbols represent affected individuals. The individuals included in the whole exome sequencing are marked with asterisk.

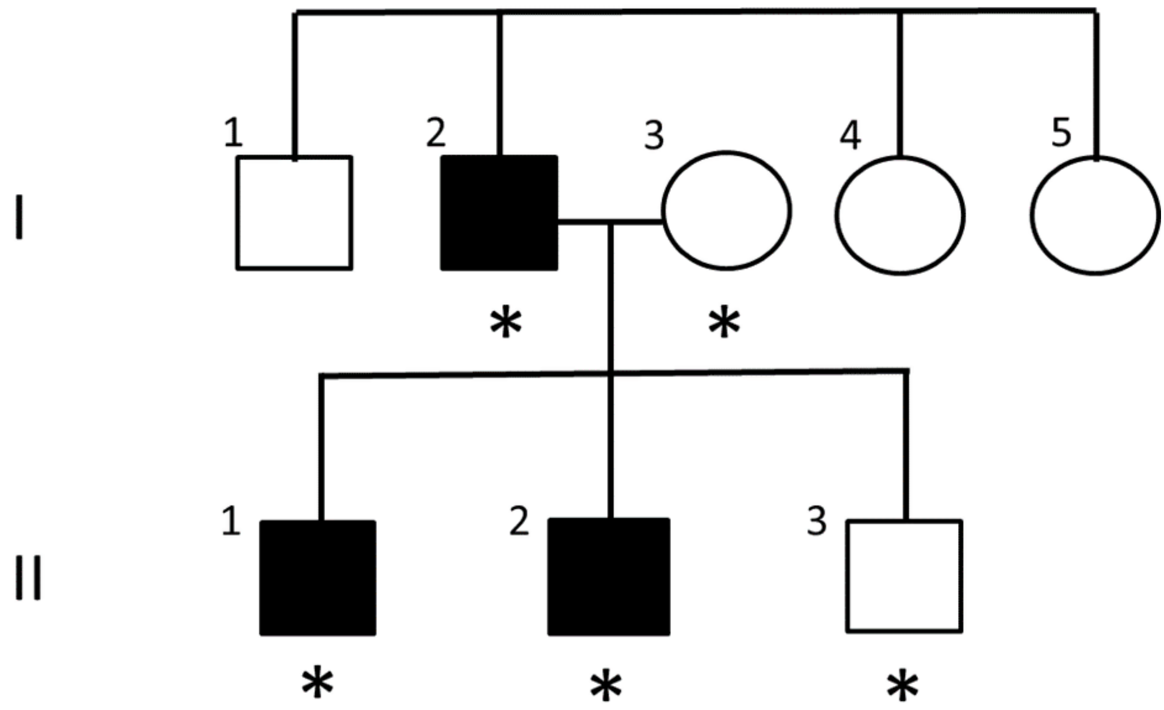

Supplemental Figure S2

X-ray images and BMD of the affected family members. A) Son II1 B) Son II2 C) Father I2

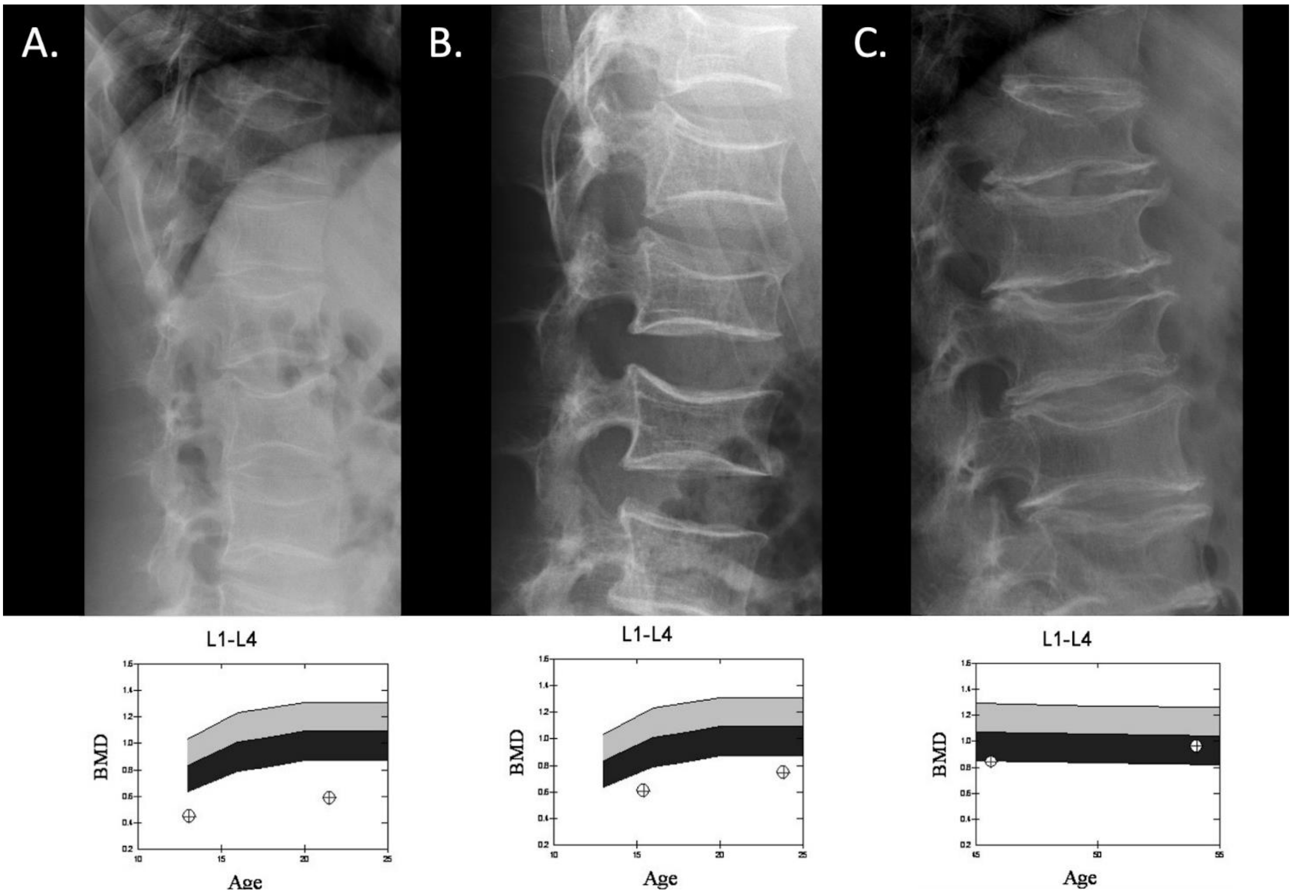

Supplemental Figure S3

Raw RPKM expression correlation among two biological replicates of Saos-2 cell controls and experiments (Saos-2 overexpressing ZNF528 or ZNF528-c.1282C>T), respectively, from Saos-2 RNA-seq data.

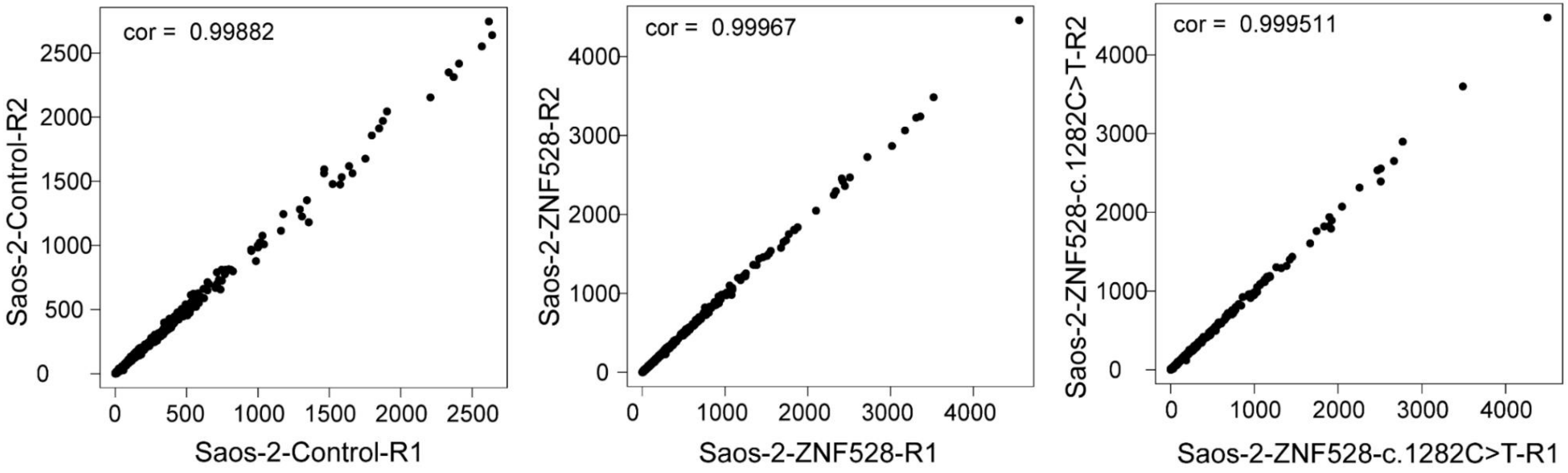

Supplemental Figure S4

Western plot showing β-actin expression for the blot presented in Supplemental Figure S4. 1=V5-ZNF528-c.1282C>T, 2=V5-ZNF528-WT, 3=Empty vector control. The 3rd replicate was used in making Figure 1C.

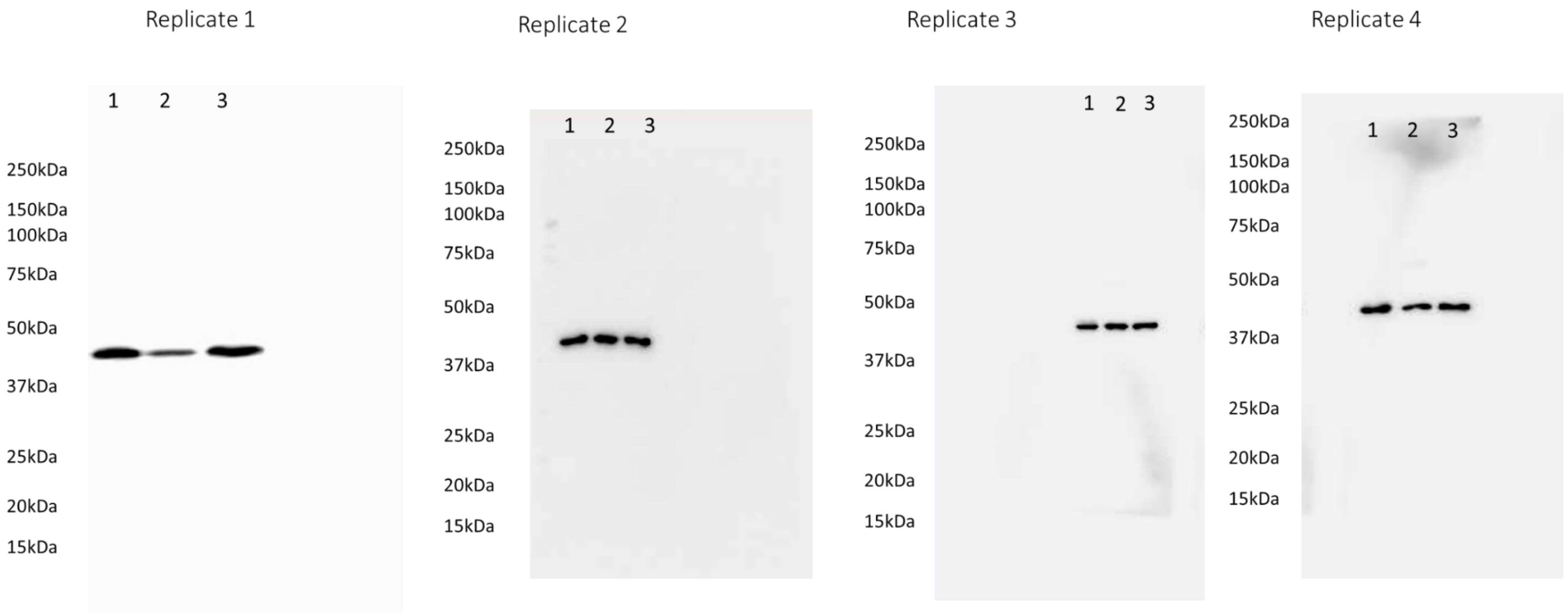

Supplemental Figure S5

Western blot showing protein expression of V5-ZNF528.1=V5-ZNF528-c.1282C>T, 2=V5-ZNF528-WT, 3=Empty vector control. The 3rd replicate was used in making Figure 1C.

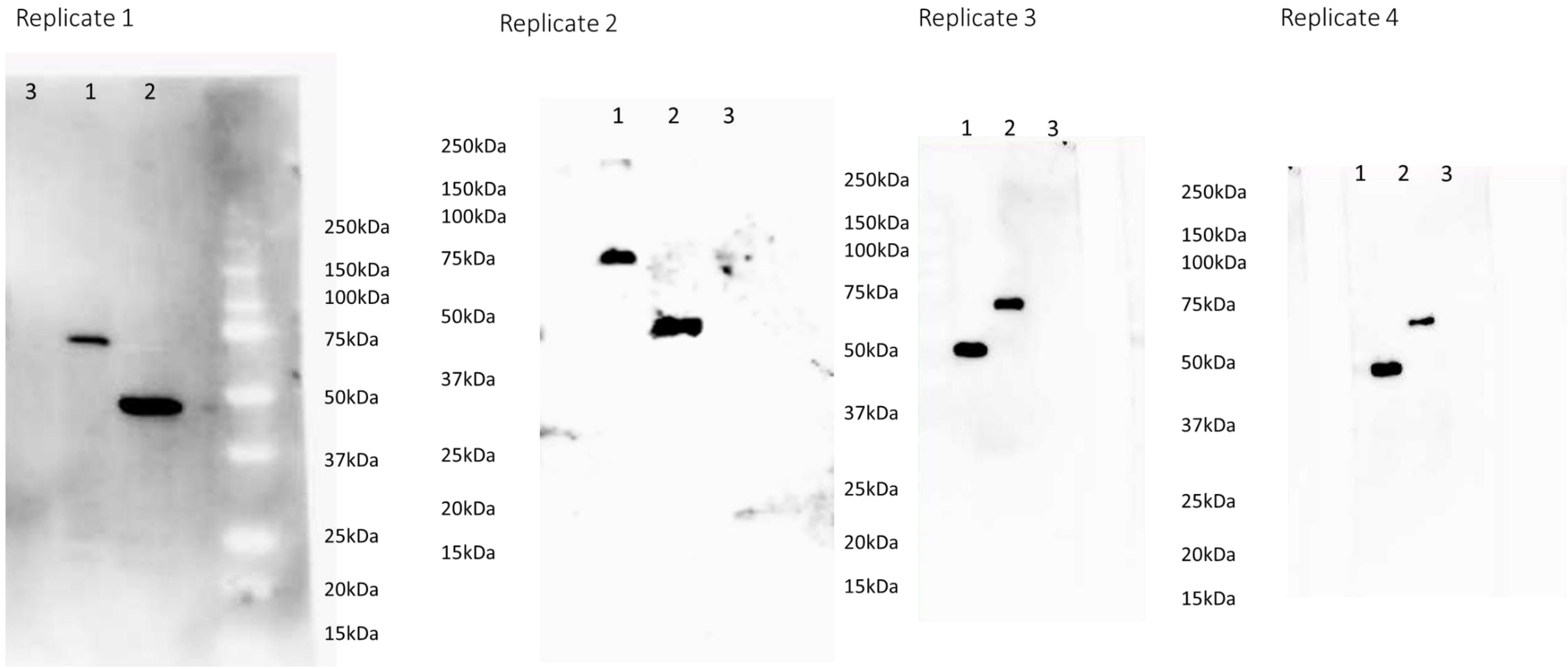

Supplemental Figure S6

V5-ZNF528-WT and V5-ZNF528-c.1282C>T expression in nucleus and cytoplasm was observed in HEK293 cells using the Western blot method.

The uncropped Western blots are presented in Supplemental Figures S7-S8.

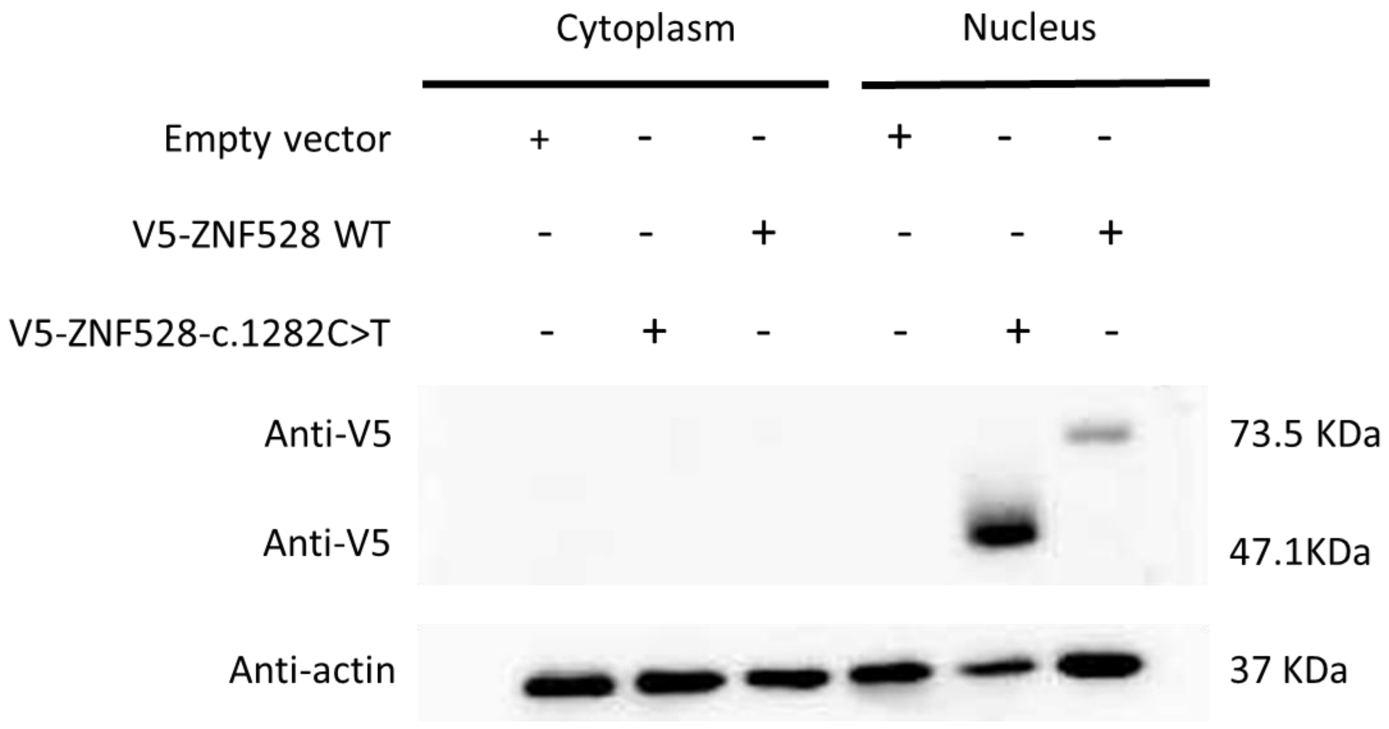

Supplemental Figure S7

Western blots showing V5-ZNF528 expression in nucleus and in cytoplasm. Replicate 2 was used in the Supplemental Figure S6. EV=Empty vector, MUT = V5-ZNF528-c.1282C>T, WT=V5-ZNF528-WT

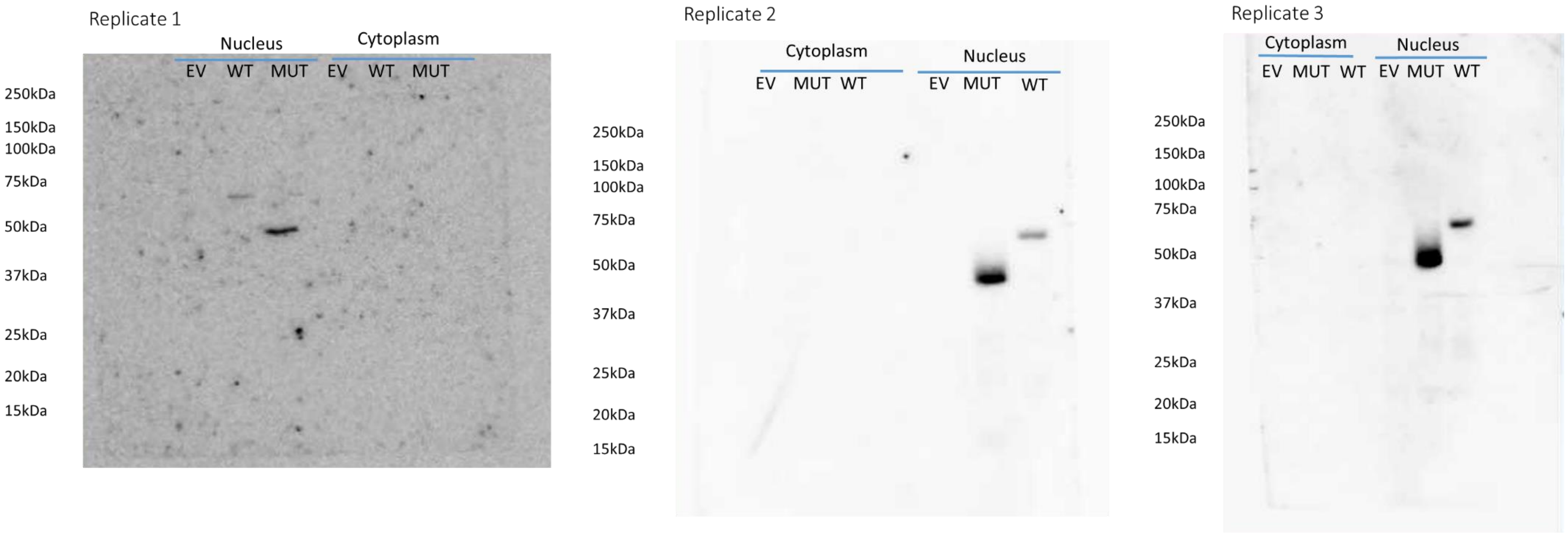

Supplemental Figure S8

Western blots indicating β-actin expression in nucleus and in cytoplasm. Replicate 2 was used in the Supplemental Figure S6. EV=Empty vector, MUT = V5-ZNF528-c.1282C>T, WT=V5-ZNF528-WT

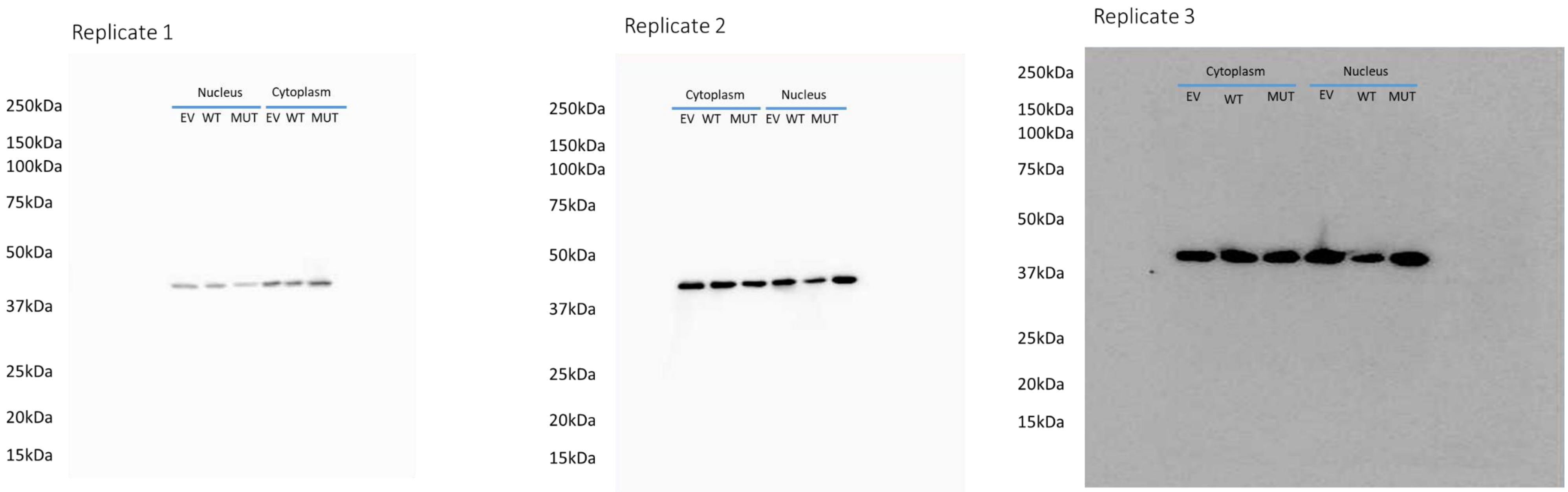

Supplemental Figure S9

Absorbance measurements of the cell viability assay. Absorbance was measured at 450 nm.

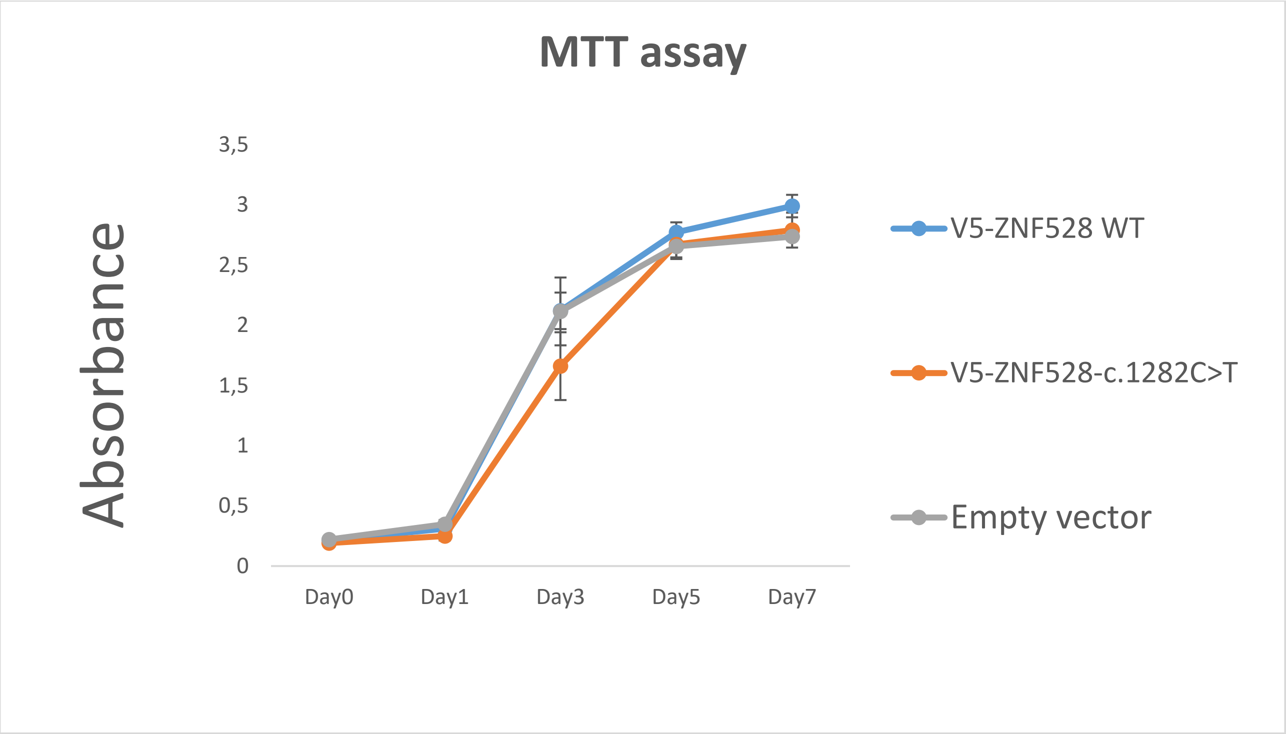

Supplemental Figure S10

Results of the cell cytotoxicity assay. The fluorescence was read with 485nm excitation source and 520nm emission filter. Data was obtained from triplicate wells and statistical significance was calculated by the two-tailed Student's t test.

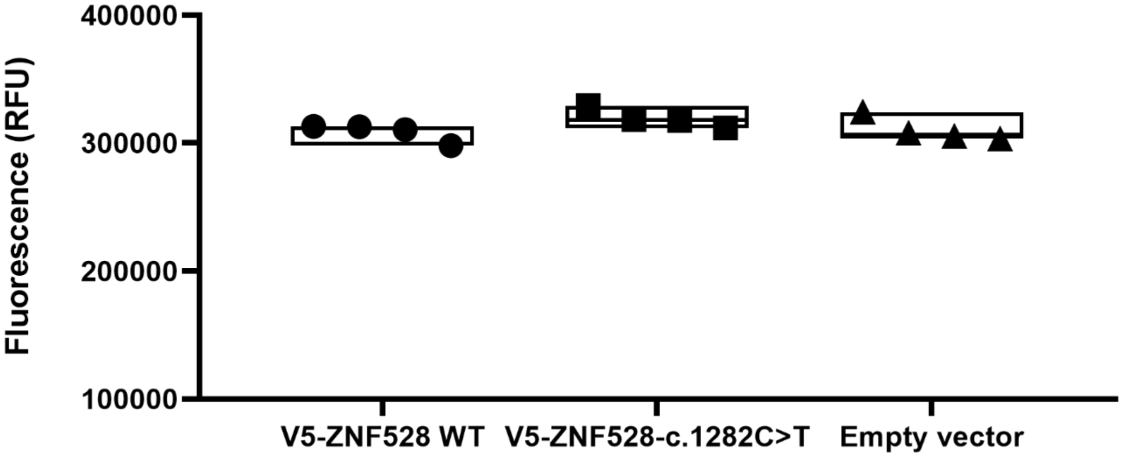

Supplemental Figure S11

Functional annotation of unique ZNF528-WT (A) and ZNF528-c.1282C>T (B) ChIP-seq peaks. Mouse phenotypes' ontology category contains data of mouse genotype–phenotype associations, and the x-axis values (in logarithmic scale) corresponds to the binomial raw (uncorrected) p-values.

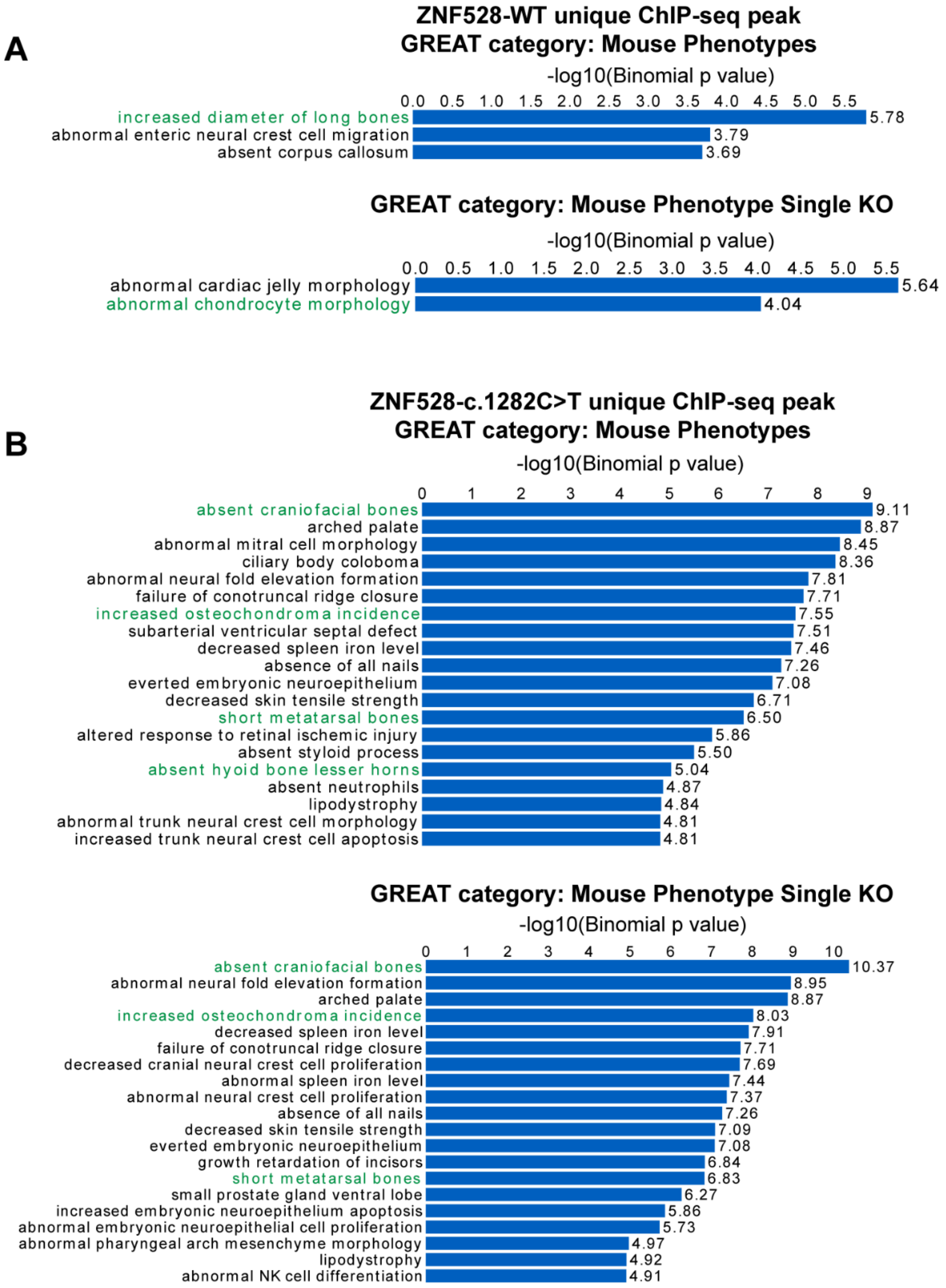

Supplemental Figure S12

Pathway enrichment analysis for ZNF528-WT unique target genes and statistical significance for osteoporosis genes. (A) Wikipathways enrichment analysis for ZNF528-WT targeted unique genes. X axis was denoted as logarithmic p value. (B) Intersection of osteoporosis genes with ZNF528-WT and ZNF528-c.1282C>T unique genes defined by RNA-Seq and ChIP-Seq. (C) Statistical significance test for the fraction of osteoporosis genes in ZNF528-WT and ZNF528-c.1282C>T by Chi-square test.

A

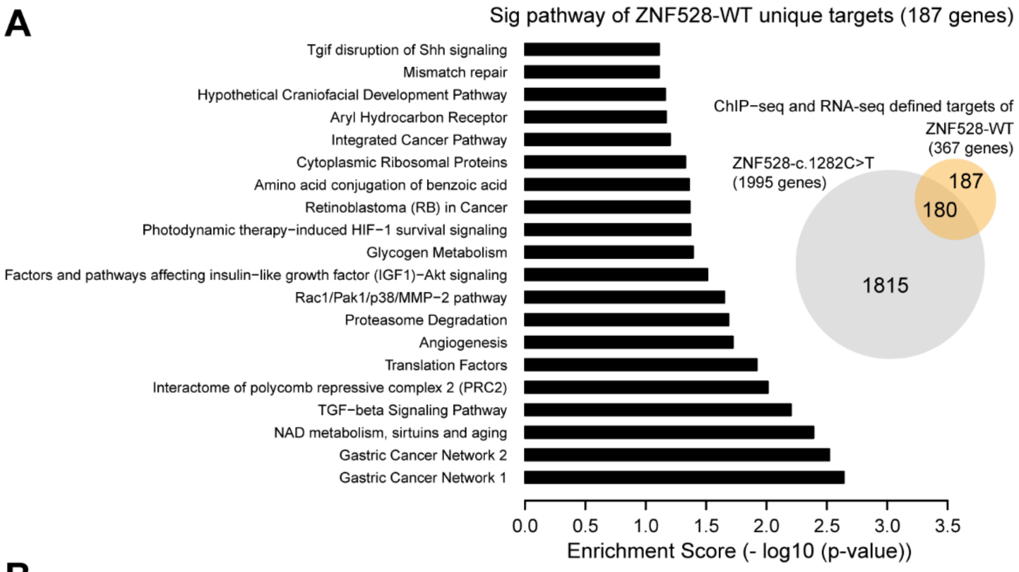

B

	Genes in total	Overlapping with osteoporosis genes	Non-overlapping with osteoporosis genes
ZNF528-WT unique target genes defined by RNA-Seq and ChIP-Seq	187	2	185
ZNF528-c.1282C>T unique target genes defined by RNA-Seq and ChIP-Seq	1815	93	1722
Common target genes of ZNF528-WT and ZNF528-c.1282C>T defined by RNA-Seq and ChIP-Seq	180	12	168

C

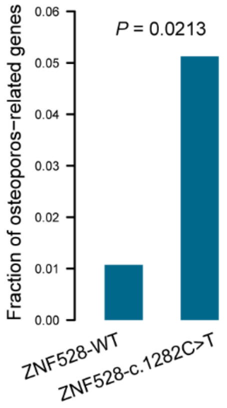

Supplemental Tables

Supplemental Table S1

Summary of the clinical information.

ID	Sex	Age (years)	BMD lumbar spine z-score	BMD proximal hip z-score	Height (cm)	Weight (kg)	Other illnesses	Medications
Family members								
I2	Male	54	-0.7	0,3	170	76	None	None
I3	Female	NA	NA	NA	NA	NA	NA	NA
II1	Male	21	-4.6	-0,9	170	64	None	None
II2	Male	23	-3.1	-0,2	170	63	None	None
II3	Male	14	-0.7	-0,1	171	62	None	None
I1	Male	67	-0.3	-0,2	179	74	NA	NA
I4	Female	67	-0.5	-0,5	160	45	Asthma, Celiac disease	NA
I5	Female	NA	NA	NA	NA	NA	NA	NA
Fibroblast cell lines								
RHH109	Male	28	NA	NA	NA	NA	None	None
RHH110	Male	56	NA	NA	NA	NA	None	None
BR1334	Male	23	NA	NA	NA	NA	None	NA

Abbreviations: NA= Not available

Supplemental Table S2

qPCR primers

Target	Primer 1	Primer 2
COL1A2	CAAGGACAAGAAACACGTCTGGCTAGGAGAAA	CAGGCGCATGAAGGCAAGTTGGGTAG
CTNNB1	TTGTGCGGCGCCATTTTAAG	TCCTCAGACCTTCCTCCGTC
CYLD	TGGCAACTGGGATGGAAGAT	CTCCTTTCCTGCGTCACACT
DKK1	TGACAACTACCAGCCGTACC	CAGGCGAGACAGATTTGCAC
ESR1	AAAGGTGGGATACGAAAAGACC	CTCATGTCTCCAGCAGACCC
JAG1	CTGCGAGCCAAGGTGTGTG	GGGTGCACTTGCGGTCTC
LFNG	CGCCACAAGGAGATGACGTT	ATACTCCACGGCCATCTTGC
LRP5	ACCGTACAGGCCCTACATCA	TGGATAGGGGTCTGAGTCCG
MEF2C	ACGATTCCGTAGGTCACAGC	GGAGGTGGAACAGCACACAA
PLOD2	GAGAAGCCCTCGAGCATCC	TGACCAAGGACCTTCACAGT
RSPO3	GGTAACCTGTGTCCCCCAAC	TCCAGACTTTTGCTGTCAGGT
RUNX2	GAACCCAGAAGGCACAGACA	GGATGAGGAATGCGCCCTAA
SOX4	CCTTTCATTCGAGAGGCGGA	GTTGCCGGACTTCACCTTCT
SOX5	CCCACGATGAGCAGAAGAA	GCTGACCTTGAACCTGGATCT
SOX9	GCTCTGGAGACTTCTGAACGA	CCGTTCTTCACCGACTTCCT
RANKL	TCCATGTTCGTGGCCCTCC	AGGATCCATCTGCGCTCTG
WLS	GAGGCTTGATTGCTCCAGGG	CCCAAGGCACGAACCATTTT
ZNF528	AGTCGCCATTCATATCTAGCAG	TGGGTTATGAGGCTGGAACG

Supplemental Table S3

Expression of *ZNF528* in bone and connective tissue cell lines.

Database	Expression
Expression Atlas	Ewing's sarcoma cell line, Bone
	Chondrosarcoma cell line, Cartilage
The Genotype-Tissue Expression (GTEx) v5	Human Cells-Transformed Fibroblasts
Cell Line Navigator	Bone cell lines (MG63, RDES, SJSA1, SW1353)

Supplemental Table S4

Results of enrichment analysis of the Najafabadi et al. 2015 data. Bone related terms are bolded.

B1hRC motifs		Trimmed *De novo* motifs		*De novo* motifs	
**GO BP**		**GO BP**		**GO BP**	
Term name	*Binom Raw P-Value*	*Term name*	*Binom Raw P-Value*	*Term name*	*Binom Raw P-Value*

hepaticobiliary system development	2.60E-11	**cranial suture morphogenesis**	4.53E-06	**positive regulation of chondrocyte differentiation**	4.24E-06
liver development	2.61E-10	**craniofacial suture morphogenesis**	1.14E-05	middle ear morphogenesis	9.83E-06
**embryonic cranial skeleton morphogenesis**	4.76E-10	negative regulation of epidermal cell differentiation	1.68E-04	regulation of cell proliferation involved in heart morphogenesis	2.78E-05
intermediate filament-based process	1.04E-08	negative regulation of epidermis development	4.14E-04	**cranial suture morphogenesis**	3.31E-05
intermediate filament cytoskeleton organization	6.79E-08	cardiac neural crest cell development involved in heart development	6.58E-04	negative regulation of epidermal cell differentiation	5.87E-05
toll-like receptor 3 signaling pathway	3.41E-07	type B pancreatic cell differentiation	8.62E-04	**craniofacial suture morphogenesis**	7.25E-05
potassium ion homeostasis	3.70E-07	cardiac neural crest cell differentiation involved in heart development	1.13E-03	metanephric nephron morphogenesis	1.10E-04
MyD88-independent toll-like receptor signaling pathway	4.03E-07			positive regulation of stem cell differentiation	1.39E-04
TRIF-dependent toll-like receptor signaling pathway	4.88E-07			desmosome organization	2.70E-04

**endochondral bone morphogenesis**	5.81E-07		myoblast fusion	2.99E-04
hindbrain morphogenesis	2.14E-06		**cartilage development involved in endochondral bone morphogenesis**	3.81E-04
toll-like receptor 5 signaling pathway	2.37E-06		negative regulation of epidermis development	4.14E-04
toll-like receptor 10 signaling pathway	2.37E-06		positive regulation of heart rate by epinephrine-norepinephrine	7.37E-04
embryonic digestive tract morphogenesis	1.87E-05		glomerular visceral epithelial cell differentiation	1.10E-03
cerebellar cortex formation	2.74E-05		embryonic foregut morphogenesis	1.24E-03
**positive regulation of chondrocyte differentiation**	3.50E-05		glomerular epithelial cell differentiation	1.35E-03
**positive regulation of BMP signaling pathway**	4.03E-05		positive regulation of the force of heart contraction by chemical signal	1.39E-03
nodal signaling pathway	1.23E-04		foregut morphogenesis	1.67E-03
auditory receptor cell differentiation	1.87E-04		positive regulation of blood pressure by epinephrine-norepinephrine	2.99E-03

nose development	3.13E-04				
**Mouse Phenotype**		**Mouse Phenotype**		**Mouse Phenotype**	
Term Name	*Binom Raw P-Value*	*Term Name*	*Binom Raw P-Value*	*Term Name*	*Binom Raw P-Value*
**arrest of tooth development**	1.15E-14	**abnormal metatarsal bone morphology**	7.09E-08	abnormal lens induction	8.26E-07
**decreased cranium height**	8.56E-08	**abnormal tendon morphology**	9.90E-08	**abnormal metatarsal bone morphology**	4.62E-06
**abnormal metacarpal bone morphology**	2.45E-07	**decreased trabecular bone thickness**	5.05E-07	**abnormal tendon morphology**	5.84E-06
**chondrodystrophy**	5.44E-07	abnormal lens induction	1.91E-06	**small limb buds**	9.82E-06
abnormal Purkinje cell differentiation	6.60E-07	decreased gamma-delta T cell number	5.20E-05	**increased vertebrae number**	3.85E-05
**abnormal metatarsal bone morphology**	7.77E-07	abnormal cerebellum posterior vermis morphology	1.40E-04	abnormal midbrain-hindbrain boundary morphology	4.95E-05
**short metatarsal bones**	2.25E-06	increased mean corpuscular hemoglobin	1.76E-04	abnormal ureter smooth muscle morphology	5.30E-05

abnormal PP cell morphology	5.37E-06	abnormal midbrain-hindbrain boundary morphology	2.53E-04	abnormal cochlear nerve compound action potential	1.01E-04
failure of palatal shelf elevation	6.24E-06	abnormal cerebellum vermis lobule morphology	5.58E-04	abnormal ischium morphology	1.52E-04
wavy neural tube	8.77E-06	bile duct proliferation	7.58E-04	conotruncal ridge hypoplasia	2.99E-04
abnormal lens induction	2.73E-05	abnormal PP cell morphology	1.18E-03	**short frontal bone**	5.92E-04
hyperresponsive to tactile stimuli	5.04E-05	calcified joint	1.23E-03	**calcified joint**	1.23E-03
increased susceptibility to noise-induced hearing loss	5.04E-05	bleb	1.82E-03	**short metatarsal bones**	1.57E-03
spinning	5.15E-05	decreased circulating noradrenaline level	2.28E-03	**absent femur**	2.23E-03
abnormal mesenchyme morphology	5.82E-05	abnormal melanoblast morphology	2.57E-03	non-pigmented tail tip	2.64E-03
abnormal foregut morphology	5.98E-05	anterior iris synechia	2.61E-03	decreased cochlear nerve compound action potential	2.82E-03
common atrium	8.44E-05	**increased presacral vertebrae number**	2.88E-03	absent tail	3.31E-03

abnormal bile duct morphology	1.15E-04				
abnormal cochlear hair cell physiology	1.24E-04				
decreased hepatocyte proliferation	1.46E-04				
**Human Phenotype**		**Human Phenotype**		**Human Phenotype**	
Term Name	*Binom Raw P-Value*	*Term Name*	*Binom Raw P-Value*	*Term Name*	*Binom Raw P-Value*
**Abnormality of the pubic bones**	3.62E-06				
**Aplasia/Hypoplasia of the pubic bone**	3.75E-05				

Abbreviations: GO BP=Genome ontology Biological Process

Supplemental Table S5

Genes identified in the pathway enrichment analysis for RNA-seq and ChIP-seq data (related to Supplemental Figure 12S B).

ZNF528-WT unique target genes	Gene name
Osteoporosis related genes (N=2)	*LBR, CABIN1*
Other genes (N=185)	*ADAM10, ANGPT1, SLC25A4, ANXA5, ARNT, KLF9, CCNG2, CDH11, CDKN3, HAPLN1, DPYD, ECT2, MEGF9, EIF2S1, EPS15, GRIK4, ARHGAP35, GUCY1A2, GYG1, HLA-H, HMGB1, ID3, IDS, ING1, EIF3E, ITGB1, KCNH1, KPNA1, IPO5, SMAD2, MAN2A1, MEF2A, MLH1, MYC, NDUFA4, NPTX2, PPIC, PPP1R8, PPP2R1B, PKN2, PSMB5, PSMC5, PTPRJ, RARB, RBBP7, ROS1, RPL11, RPL39, RPS29, SIM1, SLC1A4, SNTB1, TBX15, TFAM, TLE4, XPNPEP1, ZNF79, LUZP1, NUP214, SSPN, BCAR3, B4GALT4, EBAG9, SLC16A7, SRSF11, ARHGAP29, SEC22B, PREPL, PDE4DIP, CEP135, PHF14, CKAP5, ZBTB24, FRY, MBNL2, TRIB1, RCAN2, STAG1, PAIP1, ARPP21, RAB10, RAPGEF4, KRR1, BTN3A2, MTF2, PPM1E, ENPP4, ANKRD6, TMCC1, MON2, KDM4C, MAST2, TSPYL4, FNBP4, LARP1, SSBP2, OSBPL3, SLITRK5, TES, AK5, MMADHC, USP25, HILPDA, HN1, ZNF639, ACP6, FAM198B, NCKIPSD, VPS54, PELO, GAR1, BCAS3, TBC1D8B, CDKAL1, OXR1, PRMT6, RHOT1, CDV3, FAM46A, VPS50, DOCK10, ZNF407, LRRC40, SYBU, VAC14, C1orf112, ACSS2, ZC4H2, KIF15, ARFGEF3, HACE1, SHROOM3, RBM26, ATL2, GNPNAT1, TMEM237, TRAK2, DCTPP1, BHLHE41, GCC1, ZYG11B, CSPP1, ZNF767P, RPF1, C1orf21, ANP32E, EIF2A, DTNBP1, ZNF644, RELT, KLHL13, ACBD5, MTDH, EFHC1, GBP5, SPIN4, MACROD2, E2F7, LACC1, EFCAB3, FAM78B, TTL, LINGO2, ZNF782, TMTC3, C18orf54, DCLK2, ZNF800, DHX36, TXLNA, LCORL, NAALADL2, COL24A1, GK5, ZNF326, TRIM59, CHSY3, ZNF678, EML6, CASC15, GOLGA8B, C4orf47, MROH1, FAM160A1, LVCAT1*
**ZNF528-c.1282C>T unique target genes**	
Osteoporosis related genes (N=93)	*AGT, ANXA2, AOX1, AR, RHOA, BCL2, BMP2, BMP6, RUNX3, CD44, CNR1, CPE, CSF1, CTSB, CTSL, CTSS, TSC22D3, LPAR1, EGFR, FRZB, GALNT3, GGCX, GSK3B, GSN, HELLS, HIF1A, HSPA8, HSP90AA1, IBSP, ID4, IDH2, IGF1R, IGFBP3, IGFBP5, IL6R, CXCL8, JUN, ANOS1, LIF, LIFR, LMNA, LRP5, SMAD6, MMP2, NF1, SERPINE1, PLS3, PLXNA2, PPARA, PPARG, PPIB, PTCH1, PTGER4, PTN, PTPN1, PTPN11, RSU1, ATXN1, SH3BP2, SPARC, SPP1, SPTBN1, STAT3, THBS1, TLR4, TXNRD1, VCL, NRIP1, RECK, OGT, PDE8B, USP8, HS6ST1, WDR1, HDAC5, SPRY1, CAP1, SEMA4D, KHDRBS1, ADAMTS5, DKK1, KDM4B, SIRT1, ZBTB20, PLEKHO1, VPS53, SOX6, ANKH, MEPE, TWSG1, ARHGAP31, HIVEP3, VPS13B*
Other genes (N=1722)	*AARS, ABCA1, ACACA, ASIC2, ACLY, ACTC1, ACTN4, ACTN1, ADARB1, ADCY7, ADCY9, ADPRH, PARP4, ADRA1D, ADRA1B, AP2B1, AGA, AGL, AIM1, AK4, ALCAM, AKR1B1, AMFR, AMPH, ANK1, ANXA1, ANXA6, APBB2, BIRC3, BIRC5, APLP1, AQP9, ARF6, ARG2, RHOB, RHOC, RND3, ARSA, STS, ASNS, ASTN1, ZFHX3, RERE, ATP1A1, ATP1B1, ATP1B3, ATP2A2, ATP2A3, ATP2B4, AXL, BACH1, BARD1, BCAT1, BCL3, BDNF, BMP3, BMPR2, BNIP3, BRCA2, ZFP36L2, BTD, TSPO, CAPN5, LDLRAD4, PTTG1IP, TMEM50B, DDR1, CACNB4, CAD, CALD1, CALU, CANX, CAPN2, CAPZB, CARS, CAV1, CAV2, RUNX1, RUNX1T1, SERPINH1, CCNH, CCNT2, CD86, TNFRSF8, CD36, CD63, LRBA, CDC25B, CDC25C, CDH2, CDK8, CDKN2B, CDKN2C, CLGN, CEBPD, CEBPG, CENPE, CFL2, CTSC, RCC1, FOXN3, CHM, CHRM2, CKB,*

	CKS2, CNN3, CNTN1, COL3A1, COL5A1, COL6A1, COL6A2, COL8A1, COL10A1, COMT, KLF6, COX6A1, CRKL, VCAN, SLC25A10, CST3, CTGF, CTH, CTNNA1, CTSD, CUX1, CYB561, DAB2, DAG1, DBT, DDIT3, DDX3X, DDX5, DDX11, DHCR24, CYB5R3, NQO1, DLD, DLG1, DMD, DYNC1H1, DYNC1I1, DOCK2, DPYSL3, DRD2, RCAN1, EPYC, DUSP3, S1PR3, EEF1A1, EFNB2, EGR1, EPHA2, ENG, ENO2, STOM, EPHA3, EPHA5, EPHB3, EPRS, ERBB4, ETS1, ETV5, EVC, EXT1, EYA2, EZH2, F3, ACSL3, BPTF, FAT1, FBLN2, FBN1, FBN2, EFEMP1, FDFT1, GPC4, FGF13, FGFR2, FHL1, FOXD1, FOXO1, FOXO3, FLG, FMR1, FNTA, GALC, GALNT2, GARS, GBP2, GCNT1, GDNF, GFI1, GFPT1, GJB2, GCLC, GLI3, GLS, GNA12, GNB1, GNG11, GOLGA4, GOLGB1, GOT1, GPC1, GPI, GRID2, CXCL1, GRSF1, GSTA4, GTF2I, GZMB, HSD17B10, HTT, HIVEP1, HIVEP2, HK1, HK2, HLA-E, HMGB2, HMGCS1, NR4A1, HNRNPC, HNRNPH1, HPCAL1, AGFG1, PRMT2, PRMT1, HSPA9, HSP90AB1, NDST1, TNC, ICA1, ID2, CFI, IFNAR1, IFNGR1, IGFBP7, CYR61, RBPJ, IL1R1, IL1RAP, IL7R, TNFRSF9, IMPDH2, INCENP, INHBA, INHBB, ITGA6, IRAK2, IRF5, ITGA4, ITGB2, ITGB4, ITPR1, KCNG1, KCNN3, KIF3C, KIT, KPNB1, KRAS, KRT8, LAMB1, LAMB3, LAMC1, LAMP1, LAMP2, LCP1, LDHB, LDLR, ABLIM1, LIMK2, LMAN1, LMO7, LNPEP, LOXL2, LPP, LRCH4, LSP1, LUM, MARCKS, SMAD3, SMAD7, SMAD9, MAF, MAGOH, MAN1A1, MAOA, MAP2, MAP4, MB, MDFI, MEF2C, MET, MGAT5, MID1, MKI67, KMT2A, MME, MMP15, MMP16, MMP17, MN1, ABCC1, MSH2, MSN, MSX2, MTR, MTRR, MYH9, MYLK, MYO1C, MYO5A, MYO6, MYO10, PPP1R12A, NAP1L1, NAP1L3, NCL, NDUFA10, NDUFB9, NEDD4, NEDD9, NEK2, NEO1, NFATC2, NFE2L1, NFIX, NFKB2, NFKBIA, NID1, NINJ1, NIT1, CNOT4, NPAS2, NPC1, NPM1, NT5E, NTRK2, NTRK3, ROR1, ROR2, NUCB1, NUCB2, NVL, OAZ1, ODF2, ORC1, SLC22A18, OXA1L, PAK1, PAK2, PAX7, PBX1, PCDH7, PCNT, PCOLCE, PDE1C, PDE8A, PDK3, PDK4, ENPP2, PEX1, PFKFB3, PFKP, PGM1, PGM3, ABCB4, SERPINB6, SERPINB8, SERPINI1, PIK3CD, PIK3R1, PIK3R2, PI4KA, PLA2G4A, PLAU, PLCG1, PLRG1, PLSCR1, PLXNA1, PMAIP1, UBL3, PODXL, POLB, POLE, PPAT, PPP3CB, PRIM2, PRKAB2, PRKAR2B, PRKG1, PRKG2, MAPK9, PROX1, LGMN, TMPRSS15, PSMB4, PSMB7, PSMD4, PSMD5, PSMD12, PTEN, PTGFRN, PTGS2, PTK2, PTK7, QSOX1, PTPN13, PTPN14, PTPRA, PTPRB, PTPRD, PTPRM, PTPRZ1, RAD1, PXN, PYGB, QDPR, RAB2A, RAB3B, RAB4A, RAB6A, RABGGTB, RAP1B, RAP1GDS1, RASGRF2, KDM5A, RBBP5, RBBP6, RBBP8, RBL2, RBM3, RBMS2, RFC3, RFX5, RLF, ROBO2, RP2, RPE, RPL15, RPL38, RPLP1, RPN1, RPS6, RPS6KA3, RRBP1, RRM1, RTN1, RXRA, RYK, S100A6, S100A10, SALL1, SARS, SC5D, SDC2, SDHA, SDHB, SDHC, SFRP2, SRSF4, SRSF5, SRSF6, SGCD, SGK1, ITSN1, ST3GAL1, ST3GAL3, SKI, SLC1A5, SLC2A1, SMTN, SLC5A3, SLC6A6, SLC8A3, SLC9A2, SLIT3, SMARCA1, SMARCA2, SNAPC1, FSCN1, SNRPD1, SNTA1, SOD1, SOS1, SOS2, SOX4, SOX5, SP1, SP3, SPTAN1, SPTBN2, SRI, SRF, SRP9, SRP14, SRP68, SSFA2, SSR1, SSR2, SSTR2, SS18, STC1, HSPA13, ELOVL4, STRN, TACC1, TARS, TBL1X, TBX3, TCF7, TCF7L2, TCF12, DYNLT3, TEAD1, TFPI, TG, TGFBR2, THBS2, TIMP3, TJP1, TLE1, TLE2, TLR3, TRAPPC10, TNFAIP3, TOP1, TPP2, TRAF1, TRIO, TRPC1, TRPC4, TSPYL1, TTC3, TUBB2A, UBE2D2, UBE2H, UGCG, COL14A1, USP1, UTRN, VDAC3, VSNL1, CLIP2, WRB, XPO1, XRCC2, YES1, YWHAE, ZNF7, CNBP, ZNF37A, TRIM25, VEZF1, ZNF195, PRDM2, DAP3, ALMS1, FZD5, MANF, ADAM12, PTP4A2, ST8SIA2, TAF15, USP9X, RBM10, SMC1A, ARID1A, DYSF, COLQ, TRRAP, PICALM, AXIN1, EOMES, FZD6, FZD7, PIP5K1A, SPARCL1, CUL4B, ATRN, TPST1, SMARCA5, CDC42BPA, PPFIBP2, DGKD, PPFIA3, YARS, PDXK, CASK, RUVBL1, KLF7, USO1, CDK13, PSMG1, UNC5C, IRS2, EIF3I, EIF3J, SLC4A4, EIF4G3, SNX4, GBF1, RNMT, CD164, MPDZ, TRIM24, IQGAP1, NRP2, NRP1, PROM1, TSC22D1, CDK5R1, ASAP2, APLN, PER2, SYNJ2, EIF2B3, PRPF4B, HERC2, HERC1, RAB29, SKAP2, ATP6V0E1, KALRN, MPZL1, MAP3K14, CH25H, SPAG9, PAPSS2, PAPSS1, CLDN12, CLDN1, LDB2, DNAJA3, INA, PRPF3, CCNE2, CBFA2T2, HGS, TMSB10, SCAF11, ARHGEF2, ZMYM4, ZMYM5, VAPB, MAGI1, NOG, DHRS3, CYTH3, ATP6V1F, KLF4, NREP, B4GALT5, VAMP3, TXNL1, KIF3B, TM9SF2, NRXN1, LIPG, TGFBRAP1, NMT2, ZRANB2, SNRNP40, FAM189A2, NTN1, ITM2B, MAP4K4, HOMER3, HOMER2, AKAP7, CHST3, AKAP5, SLC4A7, SLC4A8, ADAMTS3, ADAMTS1,

	PMPCB, NPEPPS, PTGES, TP53I11, BCAR1, CDC42BPB, AKAP12, IER2, PDIA4, NFE2L3, NCOR1, NCOR2, CELSR1, NUP155, ISG15, SH3PXD2A, MICAL2, TTLL4, IQCB1, N4BP1, CLINT1, UBE3C, CROCC, KIAA0355, AQR, SEMA3E, SECISBP2L, HDAC9, ARHGAP32, TCAF1, FAM65B, STARD8, MLEC, JADE3, TM9SF4, MTSS1, SERTAD2, TATDN2, DAZAP2, CTIF, JAKMIP2, GINS1, PJA2, OSBPL2, RHOBTB1, NUAK1, MRC2, KLHL21, SGSM2, FAM20B, IQSEC1, HELZ, RBM8A, GFPT2, SLC23A2, THRAP3, AKT3, PDCD6IP, TOM1L1, SMC4, FARSB, SCAMP3, HUWE1, ATP9A, UST, ACTR3, TSPAN5, TSPAN2, PPIF, CTDSP2, RAD50, HIPK3, ARL4A, LRPPRC, TRAP1, NAMPT, AKAP9, FARP1, RBM6, LHFP, TNK2, LRRC17, UBE4B, SPEG, TRAIP, RTN3, CITED2, CORO2B, NDRG1, BASP1, ACAA2, HAX1, FST, ZBTB18, CAP2, UNC13B, SEMA6C, SEMA3C, NEBL, LAMTOR5, TM9SF1, PRDX4, SLC19A2, CXCL13, DPYSL4, CCT4, TACC2, CDC42EP3, SH2B2, ST6GALNAC2, HEXIM1, RBCK1, POLR3G, POLR3C, CSPG5, CNPY3, POLD3, TOB2, HBS1L, PLK2, SRSF10, MTHFD2, FRS2, ALDH1L1, PPP1R13L, ME3, MAN1A2, SPIN1, RALBP1, MORF4L1, AP3M2, PDIA5, TMED10, ASCC3, FERMT2, KIF2C, TLK2, RAB31, CPSF6, ABHD2, TRIOBP, DNAJB4, PRSS23, PRDM4, RCAN3, ZWINT, C14orf1, PSIP1, BAZ2A, ZNF277, CEP250, SUPT16H, ANXA10, AKAP13, MRPL3, GALNT5, RASSF8, PADI2, GPR176, CHP1, DDX42, EXOSC8, SYNPO, NID2, ITGA11, IKZF3, MRAS, COPG1, AAK1, NTNG1, CHSY1, ICK, NLRP1, R3HDM2, SEC31A, CLSTN1, BTBD3, TRAK1, KIFAP3, ATF6, SHANK2, TPX2, DIP2C, ACIN1, KDM2A, CEP152, WDFY3, STK38L, SPEN, WDTC1, KIF21B, FNBP1, SMG1, ZHX3, NCOA6, HECW1, SWAP70, MYCBP2, VWA8, ERC1, SIPA1L3, KIF1B, CDK19, TNRC6B, DCUN1D4, TBC1D9, WDR43, SNX13, GGA3, MPRIP, GPD1L, Sep-08, CYFIP1, GANAB, MDN1, GSE1, ATP11B, FAM168A, TBC1D1, RRP12, SYNE2, NUP210, VPS13A, SEL1L3, PHLPP1, ANKRD28, ANKRD12, KAZN, MTCL1, DENND5A, CAMTA1, MCF2L, DNMBP, CLEC16A, POFUT2, FBXW11, ANKS1A, BICD2, NEMP1, DNAJC13, TRIM2, MAN2B2, USP22, SASH1, TTC28, DPY19L1, SYNM, SYNE1, SMCHD1, UBR4, FAM189A1, PSD3, ARHGEF12, PUM2, ARHGEF18, ICE1, AHCYL2, ADNP, EXOSC2, DICER1, NCS1, SLC44A1, ANGPTL2, HEY1, MACF1, TMEM131, SPIDR, KAT6B, TNPO3, DDAH2, KLHDC2, LEMD3, ORC6, CD2AP, SSBP3, SLC7A11, TMEM2, GSPT2, FLRT2, FLRT1, FKBP8, CBY1, RAB3GAP2, RAD54B, QPCT, TTLL1, MTO1, HECTD1, NIPBL, PARM1, DCAF12, RCHY1, OLFML2B, CNOT10, TMEM158, POT1, NOL11, CLIC4, NSL1, WWTR1, HEATR5A, C2CD2, NECAP1, TSKU, C2CD3, SPATS2L, LRIG1, ACOT11, SUSD5, GLCE, ZNF451, SIPA1L1, LTN1, DNM3, RAB11FIP5, ANKRD17, RAI14, SZRD1, SERBP1, NAT9, KIF26A, PHGDH, TIAM2, FBXO5, SACS, EHF, CNNM4, MYOF, INTS6, TIMM9, NUFIP1, SEC22A, RNF11, CYFIP2, DNAJC2, NPTN, ATP2C1, DIEXF, STAU2, DKK3, DENND2A, AGO2, SALL3, SERP1, COQ2, SESN1, AHDC1, SRPX2, PDLIM3, RBMS3, ZNF330, RBMX, RPS6KA6, ZNF638, GOLIM4, RABGEF1, STK39, EML4, TRIB2, ACAD9, RGCC, HIPK2, MRPL13, ATAD2, C16orf72, C1GALT1C1, SETD2, CCDC59, UBE2T, SAP30BP, MYLIP, BRD7, ABT1, OLA1, YPEL1, SEC61A1, DSE, LRP12, PSAT1, BAZ2B, ERO1A, SH3KBP1, EHD4, EHD2, AK3, NTM, CDON, EXOSC3, RRP15, GLRX2, PI15, NMD3, SH3GLB1, METTL9, GOLGA7, TRIM17, RLIM, KCTD3, RNFT1, INSIG2, DYNC1LI1, NIN, NUSAP1, CRIM1, NT5C3A, ZDHHC3, PLAC8, TAOK3, CHST15, UBR5, ZNF589, EIF3L, AIG1, PRKAG2, MAGEC2, FAM8A1, RNF138, PCYOX1, PRRX2, LIMA1, DTL, LARS, JKAMP, ZC3HC1, NT5DC3, RAB6B, NBAS, DPH5, ERGIC3, KLF13, NUB1, HECA, GPRC5B, CAB39, ERAP1, RSF1, NUP54, CLIC5, ADAM22, MIS18A, CYCS, ERRFI1, GNG2, SLC38A2, DNAJC10, SSH1, RBM27, RIN2, FBXO42, PRR13, XRN1, ANKIB1, PLEKHA5, SMOX, CCDC93, DDIT4, SDK2, ING3, DDX56, RSBN1, LRRN3, PDP1, OTUD4, ZRANB1, TET2, TRPM4, BNC2, SAMD9, VPS13C, WBP1L, SYTL2, PAQR5, PALMD, TEX10, NCAPG2, RNF43, IMPAD1, SLC41A3, C1orf109, TIPIN, FAM118A, STX17, PHIP, C17orf80, CDCA4, TRMT12, ATG16L1, ZWILCH, SUSD4, IFT57, KIF26B, RALGPS2, GPATCH2, SLC6A15, ARHGEF10L, SLC25A36, APPL2, SETD5, TMEM57, RAVER2, OGFOD1, NEIL3, ASXL2, ECHDC2, FBXW7, MNS1, SLC39A9, STRBP, CHDH, LGR4, MCM10, TNFRSF19, RBM38, PLXNA3, GALNT10, RNPC3, ANKRD10, ZNF280C, TASP1, ZDHHC7, DENND4C, GPATCH2L, YEATS2,

	PACS1, FRMD4A, CCDC88A, IPO9, NECAP2, ATF7IP, HHAT, ENAH, PARVA, AGK, UGGT2, RIOK2, DOK5, WWC3, TEX2, ZC3H15, GABRQ, KMT2E, GNG12, KIAA1217, YLPM1, GPCPD1, RPRM, MUC13, C21orf59, JPH1, RAD18, ANKS1B, PNO1, SPIRE1, SMARCAD1, MCCC1, PMEPA1, OLFML3, C14orf132, TULP4, CTNNBIP1, SDHAF3, RSRP1, PLSCR4, ENTPD7, CAMK1D, GOPC, NUP107, MAN1C1, APMAP, RTN4, SMURF1, PHTF2, ASPHD2, ZMIZ1, ANKRD50, RALGAPA2, LYRM2, PBXIP1, BIRC6, ERMN, GRAMD1B, PLEKHG1, KIAA1211, ESYT2, ARID1B, HEG1, MKL2, HECW2, SRGAP1, TBC1D14, KLHL42, TAOK1, NCEH1, SIPA1L2, PREX1, TMEM181, ARHGAP21, RIC1, BEGAIN, FNIP2, USP36, SLAIN2, KIAA1462, EP400, ZSWIM5, KIAA1549, RNF213, ALS2, CACHD1, TNRC6C, USP37, CCDC181, GRHL3, TP53INP2, ENOPH1, KMT2C, MID1IP1, SNX6, NTN4, PLEKHA1, PLEKHA2, AFAP1, SAV1, RINT1, SCOC, ZFAND3, ABHD4, UBE2O, PIEZO2, CLSPN, KIF13A, FIGNL1, TSPYL2, XYLT1, NCAPG, NOC3L, NSD1, NXN, ZNF106, HHIP, MRPS25, MFSD1, SMURF2, TNS3, CREB3L2, ENGASE, ISL2, USP46, FAM129B, DCLRE1B, NABP1, PCDH20, MRPL1, PINK1, REEP1, RAPH1, NBEAL1, UPF3B, WNK3, PHACTR4, GRAMD3, DDRGK1, C11orf95, AUNIP, DSCC1, CHAC1, RNF26, MAPKAP1, DERL1, ZNF343, EFHD2, TMEM43, IRX1, RNF219, FAM184A, FAT4, ARSJ, RHBDF2, BEND5, ICE2, SMC6, MORC4, AAGAB, SUV39H2, SH3D21, TTLL7, ZFHX4, HHIPL2, PTCD2, ARHGAP28, QSER1, PEAK1, RPAP2, MAP9, RIN3, ZNF672, PRR5L, CYBRD1, NUP85, SYNDIG1, ERMP1, ELOVL7, PANK2, PGAP1, ATF7IP2, UXS1, CHD9, CCDC92, SCUBE1, FER1L4, GKAP1, CCDC68, PRR3, APOL6, APOL3, SETD7, COLEC12, SLC38A1, TMX1, PDRG1, APOLD1, LBH, UNC93B1, TRMT1L, VMP1, C6orf62, VANGL1, SBF2, TCF7L1, ABHD11, RAB33B, GLT8D2, DDX59, TMEM47, CCDC3, DYNLRB1, TLN2, SESN2, CD99L2, CRISPLD2, ESYT3, TMTC1, HMCN1, FGFBP2, NCALD, FAM172A, EMILIN2, MND1, FAM161A, PPP1R1B, RPF2, ARID5B, KIAA1109, ANTXR1, CHD6, TMEM164, MEX3B, ZCCHC9, C2orf88, FAM213A, DDI2, NIFK, HOOK3, C15orf48, RAB11FIP4, MAK16, MAP1LC3A, SLC12A8, COL25A1, TUBB6, ST6GAL2, FNDC1, SLITRK2, FAM126A, MYO18B, PRRC2B, B3GNT9, TUBA1C, POMGNT2, PLXDC2, C9orf3, ATOH8, SPRYD3, UBASH3B, TOX2, HIST1H2BK, FBF1, LRRCC1, TSPYL5, CEP295, TANC1, NAV1, MVB12B, PHLDB2, HS6ST2, SNX21, KIAA2013, ZNF160, DCAF15, MCU, SEC11C, LOC90768, FMNL3, DDX60L, BTF3L4, ANKRD44, NEXN, ELMSAN1, SNX29, SPSB4, RBM18, UBE2Q2, ADAMTSL1, ZBTB47, OLFM2, MYOCD, SYAP1, SYTL5, H2AFV, TP53INP1, MYL12B, EGLN3, CMTM7, RPLP0P2, SAAL1, CHST14, LACTB, BTBD9, SLC25A25, TMEM132B, CCDC85A, VASN, ZNF618, FAM46B, NUS1, PANX3, EXOC3-AS1, TSEN15, AGAP1, FAT3, ARL14EP, LRIG3, RHEBL1, BTBD11, TRAPPC6B, JDP2, NIPA1, TMEM170A, TBC1D16, IFFO2, SLC44A3, DNAH14, ARHGEF19, CHMP4B, MBOAT2, FAM168B, AP1S3, ACVR1C, LYPD6B, CCDC58, CHCHD4, C4orf32, IL31RA, GRPEL2, POC5, WDR36, PM20D2, SRSF12, FAM69B, GAB3, NOL4L, UBE2F, ZNF280B, SREK1, STK35, MIB2, FAM76B, LAYN, ZNF664, B3GLCT, TOM1L2, WIPF2, TMEM56, CCDC117, LINC00342, FAM117B, Sep-10, FAM84A, GPR155, CCDC80, RNF38, GLIPR2, CCDC50, IGSF11, ZNF827, SH3D19, PAQR3, THAP6, RASGEF1B, DAB2IP, TMEM161B, PLEKHG4B, SH3RF2, RNF145, AMOT, VKORC1L1, AMOTL1, ATP6V0E2, RBM33, CAMSAP1, KIAA1958, PRUNE2, OTUD7A, DENND1B, SASS6, SDE2, APCDD1L, CCDC138, THAP5, COL22A1, GLIS3, ASXL1, SYNPO2, ZNF675, PGAM5, AGO4, DNAH10, ANO6, TMEM201, SPAG17, APLF, ARL13B, RAB12, ZBTB7C, HACD2, C4orf46, RANBP3L, STK32A, KHDRBS2, ANO5, C2orf69, C3orf58, SAMD9L, CCNY, SLC16A9, MARCHF8, FAM171A1, C6orf89, RBM24, PXDC1, MTURN, KIAA1324L, SLC29A4, SEMA3D, CNOT6L, RICTOR, ZBTB38, LCLAT1, MMS22L, MSRB3, UBN2, ZDHHC23, LRRC57, RNF144B, FRMD3, NEGR1, TAB3, BOD1L1, LINC00294, OTOGL, RASSF3, B4GALNT3, HECTD4, METRNL, KCTD1, FAM102B, ZNF619, SUMF1, FAM13A-AS1, FRYL, SH3PXD2B, DCBLD1, FAM133A, CCDC85C, CCDC137, TSPAN33, ZC3H12B, FMN1, ECEL1P2, TUBB2B, SERINC2, FAM131C, NCR3LG1, KIF7, MIA3, TMEM189, INSC, TNFAIP8L3, ZNF788, FAM69A, NOTCH2NL, SNHG17, PLEKHM3, VGLL3, SAMD5, IER5L, FRRS1, HAGLR, C4orf3, HTATSF1P2, CHAC2, FBXO48, LINC00862, KIAA0754, ZFP62, FLJ32255,

	GGT8P, HHIP-AS1, PCP4L1, DIRC3, ESRG, SNHG8, POM121C, NBPF10, ZFHX4-AS1, ZBED6, MTRNR2L2, LINC00674, FGD5-AS1, MAGI2-AS3, APELA, LOC100506302, LINC00649, LINC01234, OCLN, NRAV, LOC100506730, LOC100507175, DNM3OS, HEIH, LOC101927151, LOC101927391, LINC01057, LINC01411, PIK3CD-AS2, LOC102606465
**Shared target genes**	
Osteoporosis related genes (N=12)	*APP, BMP4, COL1A2, EDN1, FYN, GJA1, GNAQ, HSPG2, IL6ST, NFKB1, NOTCH2, PLOD2*
Other genes (N=168)	*ACTA2, ADCY8, ANK3, DST, CCKAR, CDC27, CHD1, COL5A2, COL11A1, COL12A1, CTBP2, DAP, DCN, DMXL1, DDX10, DOCK3, ECE1, EPAS1, GABRE, GBE1, FOXN2, IFRD1, JARID2, KPNA2, AFF3, LGALS3BP, LSAMP, MAGEB2, ORC5, PAM, PDE4D, PHKB, PLCB4, PPP2R3A, PRKCA, REST, SATB1, SLC4A3, SLC8A1, SPOCK1, SQLE, SRPK2, NR2F2, TFDP2, TPM1, VLDLR, TRIM26, ZXDA, CDC7, FZD1, DYRK2, LGR5, CDC14A, EIF3H, GMPS, EIF2S2, WASL, PDCD5, XPR1, CD83, MAGED1, CLOCK, VGLL4, PHACTR2, TBC1D5, MAFB, PIGK, GPC6, EDIL3, ARL4C, PLXNC1, DSCR3, ANAPC10, FAM3C, PDLIM5, Sep-09, MRPS30, WDR3, DBF4, KERA, FAF1, FSTL1, AKAP11, KLF12, MGAT4A, CNKSR2, LIMCH1, PALLD, TNIK, EFR3A, ADGRL2, NEDD4L, RBFOX2, DDAH1, ORC3, MOXD1, ZZZ3, TANC2, KCNV1, MTBP, ASAP1, HP1BP3, NRN1, SNX7, LSM8, SRBD1, VPS13D, BMP2K, PNRC2, DEPDC1, TENM3, ZNF83, LMBRD1, AJAP1, CCL28, KCNQ5, UGGT1, DPYSL5, SLC39A10, NIPAL3, VANGL2, TENM2, KIDINS220, KLHL8, LRRN1, PTBP2, SLC25A19, SPCS3, PAPPA2, SLC22A23, CLSTN2, KCTD15, HMGN5, BORA, FRAS1, DDHD1, ITFG1, FAM107B, CDCA7, PLEKHA8, LTV1, ZC3H12C, SCIN, SESTD1, PXYLP1, OSBPL8, KCTD12, SSX2IP, PDZD8, ANO4, FRMD6, WTIP, B3GAT2, LINC00662, CNTN4, CNKSR3, TMEM65, ZXDB, TMTC2, DGKH, PHACTR1, WDR27, ST6GALNAC3, UBALD2, CCDC141, CCDC18, TMEM170B, LOC101927686*

Supplemental Table S6

Connective tissue related genes with ZNF528 binding motif based on Najafabadi et al. data *

Gene	Phenotype	Distance of ZNF528 binding motif to TSS [bp]
COL1A2	OI types II, III, IV	-21 896
CTNNB1	BMD, Fracture risk, WNT signaling	-568 113, -467 306, -467 077, +220 448
CYLD	NF-kappaB pathway. Osteolysis	+104 447
DKK1	WNT signaling, BMD, Fracture risk	-207 126
ESR1	BMD	+397 102
JAG1	BMD, Fracture risk	+77 240, +186 068
LFNG	vertebral column development	+937
LRP5	BMD, Fracture risk, Osteoporosis-pseudoglioma syndrome,	-9 131
MEF2C	BMD	-694 345, -694 337, -694 322, +14 476, +14 677
PLOD2	Bruck syndrome type 2	-84 033, -83 670
RSPO3	BMD	-5 411
RUNX2	Cleidocranial dysplasia	-251 531
SOX4	BMD	-917 884, -697 984, -530 334
SOX5	Kashin-Beck disease	-633 819, -517 701, -432 839, -432 649, -257 102, -42 412, +392 340, +515 055, +618 870, +619 029
SOX9	BMD, Campomelic dysplasia	-857 010, -793 260, -504 675, -90 106, +100 912, +101 929, +286 116, +290 959, +388 680
RANKL	BMD	+322 522
WLS	BMD	-181 295, -145 825, +154 625

Abbreviations: bp = base pair, TSS = Transcription Start Site

* Najafabadi HS, Mnaimneh S, Schmitges FW, Garton M, Lam KN, Yang A, Albu M, Weirauch MT, Radovani E, Kim PM, Greenblatt J, Frey BJ, Hughes TR 2015 C2H2 zinc finger proteins greatly expand the human regulatory lexicon. Nat Biotechnol 33:555-562
